# Welfare-Adjusted Life Years (WALY): A novel metric of animal welfare that combines the impacts of impaired welfare and abbreviated lifespan

**DOI:** 10.1371/journal.pone.0202580

**Published:** 2018-09-12

**Authors:** Kendy Tzu-Yun Teng, Brecht Devleesschauwer, Charline Maertens De Noordhout, Peter Bennett, Paul D. McGreevy, Po-Yu Chiu, Jenny-Ann L. M. L. Toribio, Navneet K. Dhand

**Affiliations:** 1 Sydney School of Veterinary Science, Faculty of Science, University of Sydney, Camperdown, NSW, Australia; 2 Department of Epidemiology and Public Health, Sciensano, Brussels, Belgium; 3 Department of Veterinary Public Health and Food Safety, Faculty of Veterinary Medicine, Ghent University, Merelbeke, Belgium; 4 Institute of Health and Society, Faculty of Public Health, Catholic University of Louvain, Brussels, Belgium; 5 National Taiwan University Veterinary Hospital, Taipei, Taiwan; Universidade do Porto Instituto de Biologia Molecular e Celular, PORTUGAL

## Abstract

Currently, separate measures are used to estimate the impact of animal diseases on mortality and animal welfare. This article introduces a novel metric, the Welfare-Adjusted Life Year (WALY), to estimate disease impact by combining welfare compromise and premature death components. Adapting the Disability-Adjusted Life Year approach used in human health audits, we propose WALY as the sum of a) the years lived with impaired welfare due to a particular cause and b) the years of life lost due to the premature death from the same cause. The years lived with impaired welfare are the product of the average duration of each welfare impediment, reflecting the actual condition that compromises animal welfare, the probability of an incident case developing and *impaired welfare weights*, representing the degree of impaired welfare. The years of life lost are calculated using the standard expected lifespan at the time of premature death. To demonstrate the concept, we estimated WALYs for 10 common canine diseases, namely mitral valve disease, dilated cardiomyopathy, chronic kidney disease, diabetes mellitus, atopic dermatitis, splenic haemangiosarcoma, appendicular osteosarcoma, cranial cruciate ligament disease, thoracolumbar intervertebral disc disease and cervical spondylomyelopathy. A survey of veterinarians (n = 61) was conducted to elicit impaired welfare weights for 35 welfare impediments. Paired comparison was the primary method to elicit weights, whereas visual analogue scale and time trade-off approaches rescaled these weights onto the desired scale, from 0 (the optimal welfare imaginable) to 1 (the worst welfare imaginable). WALYs for the 10 diseases were then estimated using the impaired welfare weights and published epidemiological data on disease impacts. Welfare impediment “amputation: one limb” and “respiratory distress” had the lowest and highest impaired welfare weights at 0.134 and 0.796, rescaled with a visual analogue scale, and 0.117 and 0.857, rescaled with time trade-off. Among the 10 diseases, thoracolumbar intervertebral disc disease and atopic dermatitis had the smallest and greatest adverse impact on dogs with WALYs at 2.83 (95% UI: 1.54–3.94) and 9.73 (95% uncertainty interval [UI]: 7.17–11.8), respectively. This study developed the WALY metric and demonstrated that it summarises welfare compromise as perceived by humans and total impact of diseases in individual animals. The WALY can potentially be used for prioritisation of disease eradication and control programs, quantification of population welfare and longitudinal surveillance of animal welfare in companion animals and may possibly be extended to production animals.

## Introduction

Diseases negatively affect individual companion animals in three ways, by compromising the welfare of the animal, resulting in premature death, or both. Historically, death-related statistics, such as fatality and survival time, have been used to assess the impact of a disease on affected companion animals [1–4]. However, while some diseases such as osteosarcoma in dogs [1, 5] and injection-site sarcomas in cats [6], have a high fatality rate and substantially shorten the lifespan of animals, others, such as dermatological diseases, can compromise the welfare of animals without substantially shortening their lives. These traditional, mortality-based epidemiological statistics mentioned above fail to capture the impact of these disease states on animal welfare.

Realising this, approximately three decades ago, researchers started to assess the impact of disease on welfare in companion animals [7]. Most of the instruments developed to evaluate the welfare of diseased animals focus on a single specific health condition, such as cardiac disease [8, 9], cancer [10, 11] and skin disease [12–14]. This is a weakness since multiple disorders and co-morbidities among diseases are commonly encountered in practice. Moreover, using these instruments, the comparison of the severity of welfare compromise between one health condition and another is crude if not impractical. Although some generic instruments can be used to assess the welfare of animals with different health conditions [15, 16], to the authors’ best knowledge, none has been used for quantifying the degree of impaired welfare caused by different diseases. That said, an attempt to measure the severity of canine inherited diseases was developed by Asher et al. and named the Generic Illness Severity Index for Dogs [17]. Collins et al. incorporated this index into a Breed-Disorder Welfare Impact Score to quantify the impact of some inherited diseases on dog welfare [18]. However, the four aspects of the index, namely, prognosis, treatment, complications, and behavioural impact were not weighted in the index calculation by the degree of impact on welfare.

It is apparent that, depending on the disease, a disease can adversely impact on the welfare or the lifespan of the animals, and that, for many diseases, there is negative impact on both. However, although human medicine and public health have addressed an equivalent deficit in audits of wellbeing more than 30 years ago [19], a framework that combines these two elements has not been developed for animals. Foreseeing the need to account for increasing health loss due to long-term disability caused by non-communicable diseases, such as diabetes and depression, in health measurement, the Harvard School of Public Health and the World Health Organization (WHO) launched the Global Burden of Disease (GBD) project in 1994 [20]. The GBD project introduced a new metric called Disability-Adjusted Life Year (DALY) to measure the burden of disease caused by not only premature death but also impaired health. The DALY gives a single value that denotes the loss of health caused by both disability and premature death for each of the diseases examined. The unit used in DALY is time (in years), and DALYs are the sum of the years lived with disability and the years of life lost due to premature death. The DALY considers disability as a partial loss of health and death as a complete loss of health. As the degree of health impairment differs between disabilities, *disability weights*, ranging from 0 to 1, account for this variation in DALY calculation, such that 0 means no health loss (i.e. full health) and 1 denotes complete health loss equivalent to death.

The purpose of the current study was to adapt the DALY framework to develop a novel metric, the Welfare-Adjusted Life Year (WALY), to estimate the impact of individual diseases or other causes, such as non-ideal environment or practices, in animals by considering both the impaired welfare and the premature death. We introduce the WALY specifically, in this case for dogs, as a new framework to quantify the total impact of a particular cause (an event or condition), such as a disease or a practice to animals, by combining the duration of impaired welfare and the potential life lost due to premature death caused by the same cause. We used 10 common canine diseases to illustrate how WALY can be applied. For each of the 10 diseases, veterinarians were asked to provide data that permitted estimation of the impaired welfare weights (IWWs), equivalent to the disability weights in DALY. Here we describe the approach used to estimate IWWs for the selected canine diseases. The IWWs elicited were then used to calculate WALYs for these diseases. Finally, we consider the strengths and limitations of the approaches and discuss how to improve the methodology and apply the WALY in the future.

## Materials and methods

### Welfare-adjusted life year

WALY is the sum of two components (Eq 1): (a) the years lived with impaired welfare (YLIW) due to a particular cause and (b) the years of life lost (YLL) due to premature death from the same cause.

WALY=YLIW+YLL (Eq 1)

YLIW for a given affected animal is the duration of the impaired welfare weighted by the severity of welfare compromise (i.e., IWW). YLIWs equal the sum, across each welfare impediment_*i*_, *i = 1*, *…*, *n*, of a cause, of the product of the probability (*P*_*i*_) of an incident case developing each welfare impediment (i.e., the actual physical or physiological condition that compromises the welfare resulting from the cause), with the average duration in years (*D*_*i*_) and its *IWW*_*i*_. YLLs are calculated as the standard life expectancy at age of death (*L*) (Eq 3).

$$YLIWs=\sum_{i=1}^{n}P_{i}\times D_{i}\times {IWW}_{i}$$ (Eq 2)

YLL=L (Eq 3)

To avoid simply combining impaired welfare and premature death in the equation, and in a bid to quantify and incorporate the welfare impact of not living the years lost caused by premature death into the WALY calculation, weighted YLLs, defined as the YLL value multiplied by the IWW of death, were also calculated. Weighted WALY is, therefore, the sum of YLIWs and weighted YLLs.

### Selection of diseases and welfare impediments

Based on the availability of information needed, as mentioned above, to calculate WALYs in the literature, 10 common diseases in dogs were included the current study. These were mitral valve disease, dilated cardiomyopathy, chronic kidney disease, diabetes mellitus, atopic dermatitis, splenic haemangiosarcoma, appendicular osteosarcoma, cranial cruciate ligament disease, thoracolumbar intervertebral disc disease and cervical spondylomyelopathy.

The welfare impediments were identified for each disease by consulting the literature and veterinary specialists (PB, PYC and others). A welfare impediment could be a health condition, a sign or a treatment related to the disease, that compromises dog welfare to different extents. One disease can have one or more welfare impediments. For example, dogs having mitral valve disease might have 1) “mild-moderate heart failure (with treatment)” or 2) “severe heart failure (with treatment)”, and most of them would need 3) “frequent veterinary visits (for fearful dogs)”. Each of these three is accompanied by changes that compromise dog welfare to different degrees. Note that the same welfare impediment can be found in different diseases. For example, “mild-moderate heart failure (with treatment)” and “severe heart failure (with treatment)” are welfare impediments arising from both mitral valve disease and dilated cardiomyopathy. Furthermore, although the likelihood of many welfare impediments remains similar throughout the disease course, the likelihood of others might change. Therefore, we identified key *sequelae* for each disease to allow the changes in the WALY calculation. Considerations here included the disease itself, different stages of the disease, the treatment or disease complications. Death was included as a welfare impediment for diseases resulting in mortality. A complete list of diseases, their disease sequelae and welfare impediments are shown in Table 1. Two additional welfare impediments (“severe anaemia” and “wheelchair some hours a day”) were incorporated because they are related to some welfare impediments identified. Overweight, obesity and fever were included arbitrarily. In total, 35 unique welfare impediments were considered in this study.

**Table 1:** Sequelae and welfare impediments identified for the ten diseases included in WALY study.

Disease	Disease sequela	Welfare impediment
Mitral valve disease	Mitral valve disease with signs of heart failure	Mild-moderate heart failure (with treatment)
		Severe heart failure (with treatment)
		Frequent veterinary visits (for fearful dogs)*
	Death	Death
Dilated cardiomyopathy	Dilated cardiomyopathy with signs of heart failure	Mild-moderate heart failure (with treatment)
		Severe heart failure (with treatment)
		Frequent veterinary visits (for fearful dogs)*
	Death	Death
Chronic kidney disease	Chronic kidney disease	Vomiting: two times or more a day
		Diarrhoea: two times or more a day
		Polyuria and polydipsia
		Anaemia: moderate
		Lethargy and loss of appetite
	Death	Death
Diabetes mellitus	Diabetes mellitus: pre-diagnosis	Polyuria and polydipsia
		Severe vision impairment and blindness
		Anaemia: mild
		Abdominal pain or discomfort
	Diabetes mellitus: stable phase	Frequent veterinary visits (for fearful dogs)*
		Severe vision impairment and blindness
		Abdominal pain or discomfort
		Frequent subcutaneous injections by carers at home
	Diabetic ketoacidosis	Vomiting: two times or more a day
		Diarrhoea: two times or more a day
		Lethargy and loss of appetite
		Polyuria and polydipsia
		Anaemia: mild
		Abdominal pain or discomfort
		Respiratory distress
	Death	Death
Atopic dermatitis	Atopic dermatitis	Pruritus and discomfort: mild
		Pruritus and discomfort: moderate
		Pruritus and discomfort: severe
		Frequent veterinary visits (for fearful dogs)*
Splenic haemangiosarcoma	Splenic haemangiosarcoma: pre-diagnosis	Abdominal pain or discomfort
		Cancer: lung metastasis with/without having metastasis in other parts of the body
	Cancer: diagnosis and primary therapy	Cancer: diagnosis and primary therapy
		Frequent veterinary visits (for fearful dogs)*
	Cancer: lung metastasis with/without having metastasis in other parts of the body	Cancer: lung metastasis with/without having metastasis in other parts of the body
		Abdominal pain or discomfort
		Frequent veterinary visits (for fearful dogs)*
	Death	Death
Appendicular osteosarcoma	Osteosarcoma: pre-diagnosis	Musculoskeletal problems: one limb, severe
	Cancer: diagnosis and primary therapy	Cancer: diagnosis and primary therapy
		Amputation: one limb
		Frequent veterinary visits (for fearful dogs)*
	Cancer: lung metastasis with/without having metastasis in other parts of the body	Cancer: lung metastasis with/without having metastasis in other parts of the body
		Amputation: one limb
		Frequent veterinary visits (for fearful dogs)*
	Death	Death
Cranial cruciate ligament disease	Cranial cruciate ligament disease: pre-diagnosis	Musculoskeletal problems: one limb, mild
		Musculoskeletal problems: one limb, moderate
		Musculoskeletal problems: one limb, severe
	Cranial cruciate ligament disease: recovery phase	Musculoskeletal problems: one limb, mild
		Musculoskeletal problems: one limb, moderate
		Musculoskeletal problems: one limb, severe
	Cranial cruciate ligament disease: stable phase	Musculoskeletal problems: one limb, mild
		Musculoskeletal problems: one limb, moderate
		Musculoskeletal problems: one limb, severe
Thoracolumbar intervertebral disc disease	Thoracolumbar intervertebral disc disease: pre-diagnosis	Spinal hyperesthesia: mild
		Spinal hyperesthesia: more severe
		Ataxia, paraparesis or tetraparesis
		Non-ambulatory paraparesis or paraplegia
		Urinary incontinence (upper motor neuron)
		Urinary incontinence (lower motor neuron)
	Thoracolumbar intervertebral disc disease: recovery phase	Spinal hyperesthesia: mild
		Ataxia, paraparesis or tetraparesis
		Non-ambulatory paraparesis or paraplegia
		Urinary incontinence (upper motor neuron)
		Urinary incontinence (lower motor neuron)
	Thoracolumbar intervertebral disc disease: stable phase	Spinal hyperesthesia: mild
		Ataxia, paraparesis or tetraparesis
		Non-ambulatory paraparesis or paraplegia
		Urinary incontinence (upper motor neuron)
		Urinary incontinence (lower motor neuron)
	Thoracolumbar intervertebral disc disease: reoccurrence episode	Spinal hyperesthesia: mild
		Spinal hyperesthesia: more severe
		Ataxia, paraparesis or tetraparesis
		Non-ambulatory paraparesis or paraplegia
		Urinary incontinence (upper motor neuron)
		Urinary incontinence (lower motor neuron)
	Death	Death
Cervical spondylomyelopathy	Cervical spondylomyelopathy: pre-diagnosis	Spinal hyperesthesia: mild
		Spinal hyperesthesia: more severe
		Ataxia, paraparesis or tetraparesis
		Non-ambulatory tetraparesis or tetraplegia
	Cervical spondylomyelopathy: recovery phase	Spinal hyperesthesia: mild
		Ataxia, paraparesis or tetraparesis
		Non-ambulatory tetraparesis or tetraplegia
	Death	Death

*The compromise eventuating from frequent veterinary visits reflects aversive procedures and is especially relevant for dogs that are fearful of transport and veterinary personnel, clinics and procedures.

### Estimation of the impaired welfare weights

An IWW for a welfare impediment is a number on a scale from 0 to 1 that denotes the severity of welfare compromises associated with the impediment. A value of 0 denotes the most optimal welfare that one can imagine, and a value of 1 denotes the worst welfare that one can imagine. The IWWs were derived based on the responses of veterinarians to a hard-copy questionnaire (S1 File).

Ethics approval for the procedures was granted by the University of Sydney Human Research Ethics Committee (Approval number: 2017/050).

#### Questionnaire design

Four modules were designed to be used in the questionnaire. However, as discussed below, the inclusion of three of the modules for IWW valuation depended on the version of the questionnaire administered. One module collected information about demographics of the participant, such as gender, age, graduation year, and the animal species the respondent currently worked with (dogs/cats, equines, unusual pets, livestock, captive wildlife, wildlife and laboratory/experimental animals). The other three modules contained questions based on three techniques used to evaluate the severity of the welfare impediments: paired comparison, visual analogue scale and time trade-off, respectively. Paired comparison generated preliminary IWWs for the 35 welfare impediments, a relative ranking of welfare impediments, where the resulting distances between preliminary IWWs emerge on an arbitrary scale. Using the IWW values of the same five welfare impediments generated by a visual analogue scale and time trade-off, visual analogue scale and time trade-off modules anchored preliminary IWWs onto the desired scale, 0 to 1, separately to allow comparison of the final IWW values between the two methods. The five welfare impediments selected were: “anaemia: mild”, “musculoskeletal problems: one limb, mild”, “diarrhoea: two times or more a day”, “cancer: lung metastasis with/without having metastasis in other parts of the body” and “respiratory distress”. These five were selected with the intention of covering welfare compromise across a range of severity. The questionnaire ended with a question asking if the participant had cared for their own or their family’s dogs for any of the health conditions included in the study.

Paired comparison is a process of comparing two options and judging which the respondents consider more appropriate. For the paired comparison module, each question presented two hypothetical scenarios for dogs with different welfare impediments and the participant was asked to specify which dog, in their opinion, had better welfare. A visual analogue scale is a measurement instrument that allows respondents to rate subjective characteristics or attitudes across a defined range. Visual analogue scales have been adapted for disability weight elicitation in humans with the advantage of good comprehensibility. For the visual analogue scale module, participants were first asked to rank the five selected welfare impediments according to the severity of the welfare compromise, and second asked to place them onto a scale from 0 to 100 (with zero being the worst imaginable welfare and 100 being the optimal welfare). Originally used in health economics [21], the so-called time trade-off is a technique for evaluation of quality of life under specific health conditions by measuring the length of life that participants would trade for full welfare. In the time trade-off module of the current questionnaire, the participants were able to prevent a hypothetical dog from living with one of the five anchoring welfare impediments for the next 10 years and allow it to live with optimal health and welfare. So, in exchange for optimal welfare, the dog would live for a shorter period, with the length of optimal welfare being nominated by participant depending on their opinion about the severity of the welfare compromise caused by the welfare impediment. Generally speaking, the more the condition impaired welfare, the shorter the life with optimal welfare should be lived. The participants could choose not to trade if, in their opinion, there was no difference between living with the welfare impediment described and living with optimal welfare.

#### Generation of lay descriptions for the welfare impediments

Apart from “death”, lay descriptions for each of the welfare impediments were derived from consultations with specialists (PB and PYC) and an animal welfare scientist (PM) during the questionnaire design process and were used in the questionnaires without specifying the names of the welfare impediments. Using the Five Domains animal welfare framework [22], negative welfare experiences caused by a welfare impediment were classified into one of the following domains: nutrition, environment, health, behaviour (that adopted a behavioural framework with 5 categories, namely, maintenance, elimination, locomotion, ingestion and social behaviour, proposed by Asher et al. [18]) and affective (mental) state. The physical/functional experiences said to affect at least half of the cases were used to compose the lay description with a limit of 50 words for each welfare impediment. An example of a lay description and the negative welfare experiences classified by the Five Domains is shown in Table 2 and a complete version for all welfare impediments can be found in S1 Table.

**Table 2:** An example of lay descriptions and welfare compromises classified by Five Domains of welfare impediments.

Welfare impediment	Mild-moderate heart failure (with treatment)		
Lay description	After longer periods of intense physical activity, Dog X is more likely than usual to experience tiredness, weakness and laboured breathing. As such, the dog exercises and plays somewhat less. Also, the dog sometimes coughs.		
Welfare compromise	Five Domain	Welfare compromises affecting ≥ 50% of the cases	Welfare compromises affecting ≥ 10% but <50% of the cases
	**Nutrition**	- Sodium-restricted diet is not provided.	
	**Environment**	- Cold weather and night can exacerbate the coughing.	- Hot and humid weather exacerbates the respiratory signs of heart failure.
	**Health**	- Mild to moderate heart failure - Some decrease in capacity to endure intense physical activities (e.g. exercise, running, fetching and playing) - Coughing, particularly during and after longer periods of intense physical activities - Laboured breathing after longer periods of intense physical activities, such as extended exercise and longer walks.	- Weight loss - Laboured breathing after moderate exercise or moderate walks - Ascites - Syncope
	**Behaviour**	- Undertakes somewhat fewer intense physical activities than usual - Somewhat less interaction with people, other dogs or animals than usual	- Eats less than usual; refuses regular food - Cannot sleep well due to coughing or trouble breathing during the night - Physical restriction is enforced by the carer: the dog exercises, plays and interacts with people, other dogs or animals less than usual
	**Affective state**	- Some discomfort - Appears tired and weak more easily than usual after longer periods of intense physical activities - Shortness of breath after longer periods of intense physical activities	- Some loss of appetite - Fewer positive affective states due to physical restriction than usual - Sometimes restless at night

#### Sample size and questionnaire type

Assuming the expected population standard deviation to be 25 (i.e., one-quarter of the visual analogue scale) and using the standard sample size formula to estimate a single mean, the study required a sample size of 28 to estimate a mean value for each welfare impediment on the visual analogue scale with 95% confidence and a precision of 10. Therefore, we aimed to attain a minimum of 30 responses for each welfare impediment in the paired comparison module, the visual analogue scale module and the time trade-off module. To achieve this whilst limiting the questionnaire completion time to 15 minutes, three versions of the questionnaire were designed: (A) the visual analogue scale module and 16 paired comparison questions in the paired comparison module, (B) the time trade-off module and 16 paired comparison questions in the paired comparison module, and (C) both the visual analogue scale and the time trade-off modules and 11 paired comparison questions in the paired comparison module. As mentioned above, all questionnaires contained the demographics module and the question about participants’ personal experience with certain health conditions in dogs. Questionnaires A and B were expected to be completed by 10 participants each, and Questionnaires C by 20 participants each. In total, a minimum of 40 participants were needed for this study.

#### Participants

Participants were required to be veterinarians registered in Australia or New Zealand and were recruited during the 2017 Australian Veterinary Association Annual Conference on 5^th^ and 6^th^ June 2017 in Melbourne, Australia. Each participant received an AU$20 gift card for participating in the survey. Random numbers were used to allocate the three questionnaire versions among the participants.

### Statistical analysis

Responses to the questionnaire were encoded in Microsoft Excel 2010 (Microsoft Corp. Redmond, Washington, United States), and descriptive statistics of the demographics of participants, IWW generation and WALY calculation were conducted in R version 3.3.0 (R Core Team).

#### Generation of impaired welfare weights

The paired comparison data were analysed using probit regression [23]. A pooled dataset of paired comparison questions was structured in a specific format with each question representing a row (S2 Table). The 1^st^ welfare impediment in the question was marked as 1, the 2^nd^ welfare impediment in the question marked as -1, and other welfare impediments not included were 0. A binary variable was created based on the participants’ responses at 1 if the first welfare impediment was chosen, or 0 if the second welfare impediment was chosen. Probit regression analyses were run with the participant response as the outcome variable and all the welfare impediments as dummy variables (i.e., 1 and -1).

IWWs of the five welfare impediments using the results from the visual analogue scale and time trade-off questions were calculated separately using the following formulae (Eqs 4 and 5), where IWW_VAS and_ IWW_TTO_ represent the IWWs based on the visual analogue scale (VAS) and time trade-off (TTO) questions, respectively, and *n* is the number of responses.

$${IWW}_{VAS}=\frac{\sum_{i=1}^{n}\left(1-\frac{{welfare\;impediment}_{i}}{100}\right)}{n}$$ (Eq 4)

$${IWW}_{TTO}=\frac{\sum_{i=1}^{n}\left(1-\frac{{welfare\;impediment}_{i}}{10}\right)}{n}$$ (Eq 5)

The mean IWWs were compared descriptively and using a 2-sample t-test between two categories of the binary demographics that are likely to relate to the evaluation of IWWs and had at least 20 participants in both groups. Demographics that had numeric results were grouped into binary with the median value as the cut-off value. These binary demographics included gender (male or female) [24, 25], age (<36 or ≥36), graduation year (<2005 or ≥2005) [24], special interest in small animal medicine and/or surgery (yes or no), and working with livestock (yes or no) [25, 26]. A *P*-value of <0.05 was considered to be significant.

To project the results from the probit regression model on an IWW scale ranging from 0 to 1, we ran locally weighted scatterplot smoothing (loess) regression models of the probit regression coefficients from the paired comparison valuation method versus the logit transformed IWW values from the visual analogue scale and time trade-off [27]. The span values started with 0.75 (range from 0 to 1) and, if needed, the starting span value was adjusted, to ensure model convergence. We then predicted logit transformed IWWs for each of the probit coefficients from the loess fit. To change the scale and obtain values ranging between 0 and 1, an inverse logistic transformation of these predicted IWW was applied.

The uncertainty of the predictions of the loess regression model was propagated using Monte Carlo simulations [28]. For each mapped IWW, 100,000 simulations were drawn from a logit-normal distribution specified by the predicted IWW and standard error. The resulting uncertainty distribution was summarised by its mean and a 95% uncertainty interval (UI) defined as the distribution’s 2.5^th^ and 97.5^th^ percentile [28].

#### Welfare-adjusted life year calculation

Using information from the peer-reviewed literature (Table 3), the life table from Inoue et al. [29] and IWWs elicited by our study, YLIWs (Eq 2), YLLs (Eq 3), weighted YLLs, WALY and weighted WALY were calculated for the ten diseases. To estimate the YLLs for the 10 canine diseases, the age of premature death for each disease was estimated using the average age at diagnosis and the average survival time for a disease and, using the derived age of death, YLL was estimated by implementing a linear interpolation using the life table. The proportional contributions of YLIWs and YLLs to WALYs were calculated.

**Table 3:** Peer-reviewed articles used for data extraction for the estimation of welfare-adjusted life year for 10 canine diseases on which the current study focused.

Disease	Average age at diagnosis^1^	Average survival time from time of diagnosis (years)	Probability of an incident case developing and duration of welfare impediments in disease sequelae
Mitral valve disease	Borgarelli et al., 2008 [mean: 10.6 (SD^2^: 2.62)] [32]	Borgarelli et al., 2008 [median: 1.63 (range: 0.03–6)] [32]	Borgarelli et al., 2008 [32]
Dilated cardiomyopathy	Martin et al., 2009 [median: 6.67 (IQR^3^: 4.79–8.54)] [33]	Martin et al., 2009 [median: 0.37 (IQR: 0.08–1.15)] [33]	Martin et al., 2009 [33]
Chronic kidney disease	O'Neill et al., 2013 [median: 12.39 (range: 0.75–19.14)] [34]	O'Neill et al., 2013 [median: 0.62 (95% CI^4^: 0.31–0.89)] [34]	O'Neill et al., 2013 [34]
Diabetes mellitus	Mattin et al., 2014 [median: 9.9 (range: 3.3–17.4)] [35]	Mattin et al., 2014 (median: 1.44) [35]	Hess et al., 2000 [36]; Fall et al., 2007 [4]; De Causmaecker et al., 2009 [37]
Atopic dermatitis	Saridomichelakis et al., [median: 2.5 (range: 0.17–8)] [38]	NA^5^	Rybníček et al., 2009 [39]
Splenic haemengiosarcoma	Kahn et al., 2013 [median: 9.9 (range: 7–14.1)] [40]	Estimated using information from Kahn et al., 2013 [40]	Kahn et al., 2013 [40]; Kim et al., 2007 [41]
Appendicular osteosarcoma	Bacon et al., 2008 [median: 8 (range: 1.3–13.2)] [42]	Bacon et al., 2008 [median: 0.71 (95% CI : 0.48–0.94)] [42]	Bacon et al., 2008 [42]
Cranial cruciate ligament disease	Multiple [43]	NA	Cabrera et al., 2008 [43]; Stein and Schmoekel 2008 [44]; Voss et al., 2008 [45]; Oxley et al., 2013 [46]
Thoracolumbar intervertebral disc disease	Aikawa et al., 2012 [median: 5 (range: 1.5–14)] [47]	Multiple [48]	Aikawa et al., 2012 [47]; Aikawa et al., 2012 [48]; Fadda et al., 2013 [49]; Salger et al., 2014 [50]
Cervical spondylomyelopathy	McKee et al., 1999 [median: 7 (range: 3–11)] [51]	Estimated using information from da Costa et al., 2008 [52] and de Decker et al., 2009 [53]	Fadda et al., 2013 [49]; McKee et al., 1999 [51]

^1^: Decimal place depended on the results in the articles cited.

^2^: Standard deviation.

^3^: Interquartile range.

^4^: Confidence interval.

^5^: Not applicable.

The uncertainty of YLIW, YLL and WALY was propagated using 100,000 Monte Carlo simulations and results were presented as the mean and 95% UI of the resulting uncertainty distributions using “mc2d” [30] and “FERG” [31] packages in R.

## Results

### Welfare comprise weight generation

#### Demographics of respondents

There were 61 participants in total, of whom 41 (67%) were females and 20 (33%) were males. The median age was 36 (interquartile range: 29–51; range: 24–69) years and the median time since graduation was 12 years (interquartile range: 4–28; range: 1–45). Most participants had owned dogs or grown up with dogs (90%). Thirty (49%) had a special interest in small animal medicine and/or surgery. At the time of completing the questionnaire, 18 (30%) participants worked with livestock, and only 2 (3%) did not work at all with dogs and cats. More demographics can be found in S3 Table.

Across the 61 participants, the random allocation of the three questionnaire versions resulted in completion of the demographics module and the question on participants’ personal experience with certain dog health conditions by 61 respondents, the visual analogue scale module by 45 (16 for version A and 29 for version C) and the time trade-off module by 45 (16 for version B and 29 for version C), however for the visual analogue scale and time trade-off module modules the non-feasible answers of 1 participant each were excluded from the analysis. For the paired comparison module, 828 paired comparison questions were answered by the 61 respondents excluding three questions that were not completed because two participants were unable to distinguish the severity of the welfare compromise between the two options in these questions.

#### Impaired welfare weights

The five IWWs elicited by the visual analogue scale and time trade-off followed the same severity order (Table 4) although the distribution of the five IWWs was greater in the latter than in the former. Generally, the five IWWs were higher for participants who were female, younger than 36 years, had graduated in 2005 or later, or did not work with livestock at the time of completing the questionnaire (Table 5). From the IWWs elicited using the visual analogue scale, mean IWW for “anaemia: mild” was scored significantly higher by females than by males (0.25 vs. 0.14); mean IWW of “cancer: lung metastasis with/without having metastasis in other parts of the body” was scored significantly higher by young graduates than by older graduates (0.81 vs. 0.70); “diarrhoea: two times or more a day” was scored significantly lower by veterinarians interested in small animal medicine and/or surgery than their counterparts (0.42 vs. 0.55); and mean IWW of “musculoskeletal problems: one limb, mild” was scored significantly higher by veterinarians working with livestock than by those who did not work with livestock (0.46 vs. 0.31). None of the five mean IWWs using time trade-off were statistically significant different among respondents across the various demographic categories.

**Table 4:** The impaired welfare weights (IWWs) for five welfare impediments generated by visual analogue scale (VAS) and time trade-off (TTO) ordered from lowest to highest.

Welfare impediment	IWW (VAS)			IWW (TTO)		
	Mean	SD^1^	Range	Mean	SD	Range
Cancer: lung metastasis with/without having metastasis in other parts of the body	0.759	0.156	0.30–1.00	0.858	0.166	0.30–0.99
Musculoskeletal problems: one limb, mild	0.414	0.169	0.05–0.80	0.386	0.324	0.00–0.99
Anaemia: mild	0.215	0.135	0.03–0.53	0.198	0.260	0.00–0.95
Respiratory distress	0.897	0.094	0.50–1.00	0.917	0.144	0.40–1.00
Diarrhoea: two times or more a day	0.483	0.210	0.15–0.98	0.480	0.326	0.00–0.99

Legends: IWWs range from 0 (the optimal welfare imaginable) to 1 (the worst welfare imaginable).

^1^: Standard deviation

**Table 5:** The mean impaired welfare weights (IWWs) of subgroups for five welfare impediments generated by visual analogue scale (VAS) and time trade-off (TTO).

Name	Gender		Age (year)		Graduation year		Interest in small animal medicine/surgery		Working with livestock	
Visual analogue scale	Female	Male	<36	≥36	≥2005	<2005	No	Yes	No	Yes
Cancer: lung metastasis with/without having metastasis in other parts of the body	0.78	0.72	0.79	0.73	0.81*	0.70*	0.77	0.75	0.77	0.74
Musculoskeletal problems: one limb, mild	0.44	0.36	0.42	0.41	0.43	0.40	0.39	0.44	0.46*	0.31*
Anaemia: mild	0.25*	0.14*	0.21	0.22	0.22	0.21	0.24	0.20	0.22	0.21
Respiratory distress	0.91	0.87	0.90	0.89	0.90	0.89	0.92	0.88	0.92	0.86
Diarrhoea: two times or more a day	0.50	0.45	0.46	0.51	0.48	0.48	0.55*	0.42*	0.46	0.53
**Time trade-off**										
Cancer: lung metastasis with/without having metastasis in other parts of the body	0.86	0.85	0.89	0.82	0.89	0.82	0.86	0.85	0.87	0.81
Musculoskeletal problems: one limb, mild	0.40	0.36	0.47	0.31	0.45	0.31	0.3	0.46	0.41	0.27
Anaemia: mild	0.20	0.19	0.23	0.17	0.22	0.17	0.18	0.21	0.21	0.13
Respiratory distress	0.91	0.94	0.96	0.88	0.96	0.87	0.93	0.90	0.92	0.89
Diarrhoea: two times or more a day	0.53	0.37	0.56	0.40	0.56	0.39	0.45	0.50	0.50	0.39

Legends: IWWs range from 0 (the optimal welfare imaginable) to 1 (the worst welfare imaginable).

* Significant difference between the two subgroups based on *P*-value <0.05.

**Fig 1:**
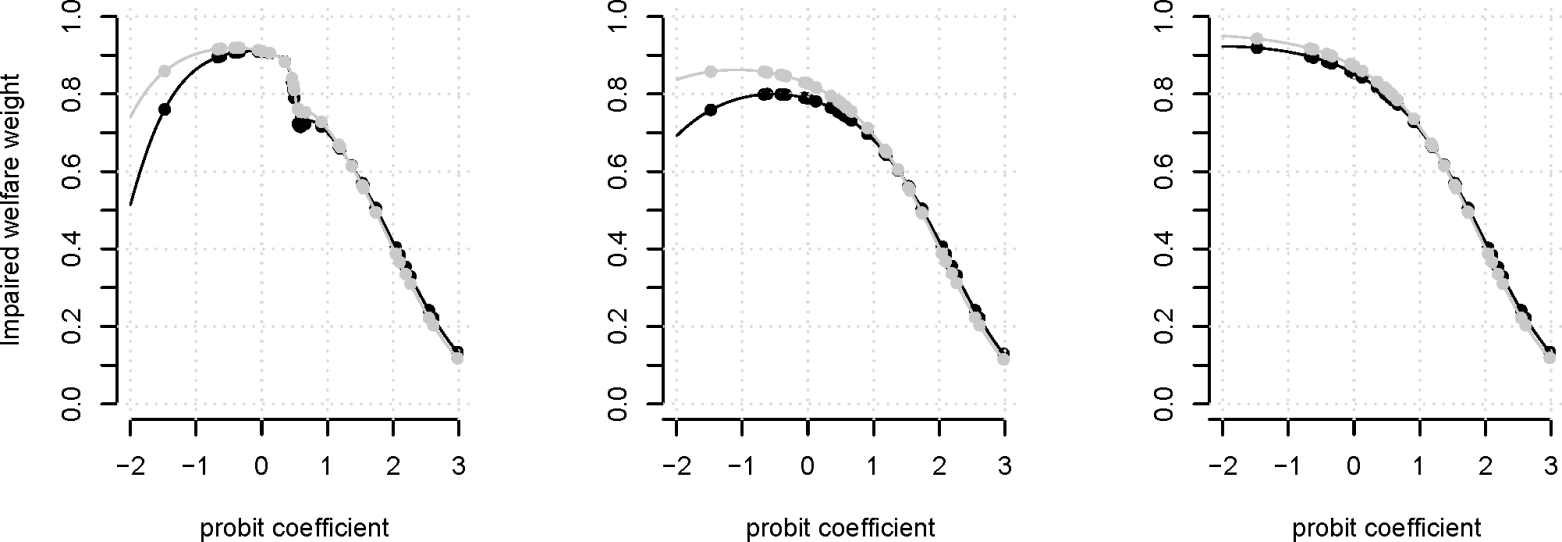
Comparison of impaired welfare weights anchored by visual analogue scale (black) and time trade-off (grey). Fig 1(a) displays the models using all the five anchoring welfare impediments, and the models in (b) and (c) excluded “respiratory distress” and “cancer: lung metastasis with/without having metastasis in other parts of the body”, respectively.

Two sets of IWWs were generated after anchoring IWWs from the visual analogue scale and the time trade-off methods, respectively (Fig 1). In theory, the ranked severity of the welfare impediment elicited by paired comparison should align with ranks generated by anchoring methods, resulting in a monotonic trend. However, “cancer: lung metastasis with/without having metastasis in other parts of the body” was rated more severe than “respiratory distress” in paired comparison but less severe in both of the anchoring methods. Therefore, additional sets of IWWs elicited using the anchoring IWWs results without welfare impediment “respiratory distress” or “cancer: lung metastasis with/without having metastasis in other parts of the body” were built separately and compared (Fig 1). IWWs elicited by loess regression without including the anchoring welfare impediment “respiratory distress” are displayed in Table 6. All the IWW results generated by the six models are displayed in S4 Table.

**Table 6:** Impaired welfare weights (IWWs) for the 35 welfare impediments.

Welfare impediment	IWW (VAS)	IWW (TTO)
Mild-moderate heart failure (with treatment)	0.357 (0.305–0.411)	0.337 (0.304–0.372)
Severe heart failure (with treatment)	0.646 (0.524–0.755)	0.655 (0.575–0.728)
Cancer: diagnosis and primary therapy	0.728 (0.599–0.833)	0.752 (0.673–0.821)
Cancer: lung metastasis with/without having metastasis in other parts of the body	0.757 (0.679–0.824)	0.857 (0.823–0.887)
Musculoskeletal problems: one limb, mild	0.387 (0.332–0.444)	0.368 (0.332–0.405)
Musculoskeletal problems: one limb, moderate	0.760 (0.637–0.858)	0.793 (0.720–0.854)
Musculoskeletal problems: one limb, severe	0.786 (0.675–0.872)	0.829 (0.767–0.879)
Severe vision impairment and blindness	0.602 (0.490–0.707)	0.604 (0.530–0.675)
Overweight	0.504 (0.420–0.588)	0.494 (0.438–0.550)
Obesity	0.406 (0.348–0.467)	0.389 (0.350–0.428)
Spinal hyperesthesia: mild	0.777 (0.661–0.868)	0.815 (0.749–0.870)
Spinal hyperesthesia: more severe	0.740 (0.612–0.843)	0.767 (0.689–0.833)
Ataxia, paraparesis or tetraparesis	0.504 (0.420–0.587)	0.494 (0.437–0.549)
Non-ambulatory paraparesis or paraplegia	0.740 (0.614–0.843)	0.767 (0.691–0.833)
Non-ambulatory paraparesis or paraplegia: on wheelchair some hours a day	0.641 (0.520–0.750)	0.649 (0.570–0.722)
Non-ambulatory tetraparesis or tetraplegia	0.795 (0.698–0.872)	0.846 (0.795–0.888)
Urinary incontinence (upper motor neuron)	0.747 (0.621–0.848)	0.776 (0.700–0.840)
Urinary incontinence (lower motor neuron)	0.783 (0.670–0.871)	0.825 (0.762–0.876)
Anaemia: mild	0.224 (0.163–0.295)	0.203 (0.165–0.247)
Anaemia: moderate	0.693 (0.565–0.803)	0.711 (0.629–0.783)
Anaemia: severe	0.795 (0.696–0.872)	0.845 (0.793–0.888)
Pruritus and discomfort: mild	0.560 (0.459–0.657)	0.556 (0.489–0.621)
Pruritus and discomfort: moderate	0.748 (0.623–0.849)	0.778 (0.702–0.842)
Pruritus and discomfort: severe	0.750 (0.624–0.850)	0.780 (0.704–0.843)
Respiratory distress	0.796 (0.713–0.865)	0.857 (0.815–0.892)
Polyuria and polydipsia	0.332 (0.282–0.386)	0.312 (0.279–0.347)
Vomiting: two times or more a day	0.796 (0.709–0.866)	0.855 (0.811–0.892)
Diarrhoea: two times or more a day	0.502 (0.418–0.586)	0.492 (0.436–0.548)
Abdominal pain or discomfort	0.741 (0.613–0.844)	0.768 (0.691–0.835)
Lethargy and loss of appetite	0.736 (0.609–0.840)	0.763 (0.685–0.830)
Fever	0.796 (0.700–0.871)	0.848 (0.799–0.889)
Amputation: one limb	0.134 (0.072–0.223)	0.117 (0.078–0.168)
Frequent veterinary visits (for fearful dogs)	0.244 (0.185–0.310)	0.223 (0.186–0.264)
Frequent subcutaneous injections by carers at home	0.555 (0.455–0.652)	0.551 (0.484–0.616)
Death	0.562 (0.460–0.659)	0.558 (0.491–0.624)

Legends: IWWs range from 0 (the optimal welfare imaginable) to 1 (the worst welfare imaginable). The two sets of IWWs were anchored by visual analogue scale (VAS) and time trade-off (TTO), respectively, after excluding the anchoring welfare impediment “respiratory distress” in the model building process.

The ranges of the IWWs for the 35 welfare impediments elicited by time trade-off (0.117–0.857) were wider than those elicited by visual analogue scale (0.134–0.796), with both excluding “respiratory distress” in the anchoring process. Only one welfare impediment, “cancer: lung metastasis with/without having metastasis in other parts of the body”, elicited rankings that differed between these two sets of IWWs. “Death” was the 23^rd^ severest welfare impediment in both sets of results, with an IWW at 0.562 and 0.558 when anchored by the visual analogue scale and time trade-off, respectively. The IWWs of most of the welfare impediments that differ only in the level of severity align with their ranking (e.g., the IWWs of “anaemia: mild”, “anaemia: moderate” and “severe anaemia” anchored by the visual analogue scale were 0.224, 0.693 and 0.795, respectively). However, “overweight” was rated as compromising dog welfare more than “obesity”, and the same phenomenon was observed for spinal hyperesthesia in that “spinal hyperesthesia: mild” was rated as compromising dog welfare more than “spinal hyperesthesia: severe”.

### Welfare-adjusted life year results

**Fig 2:**
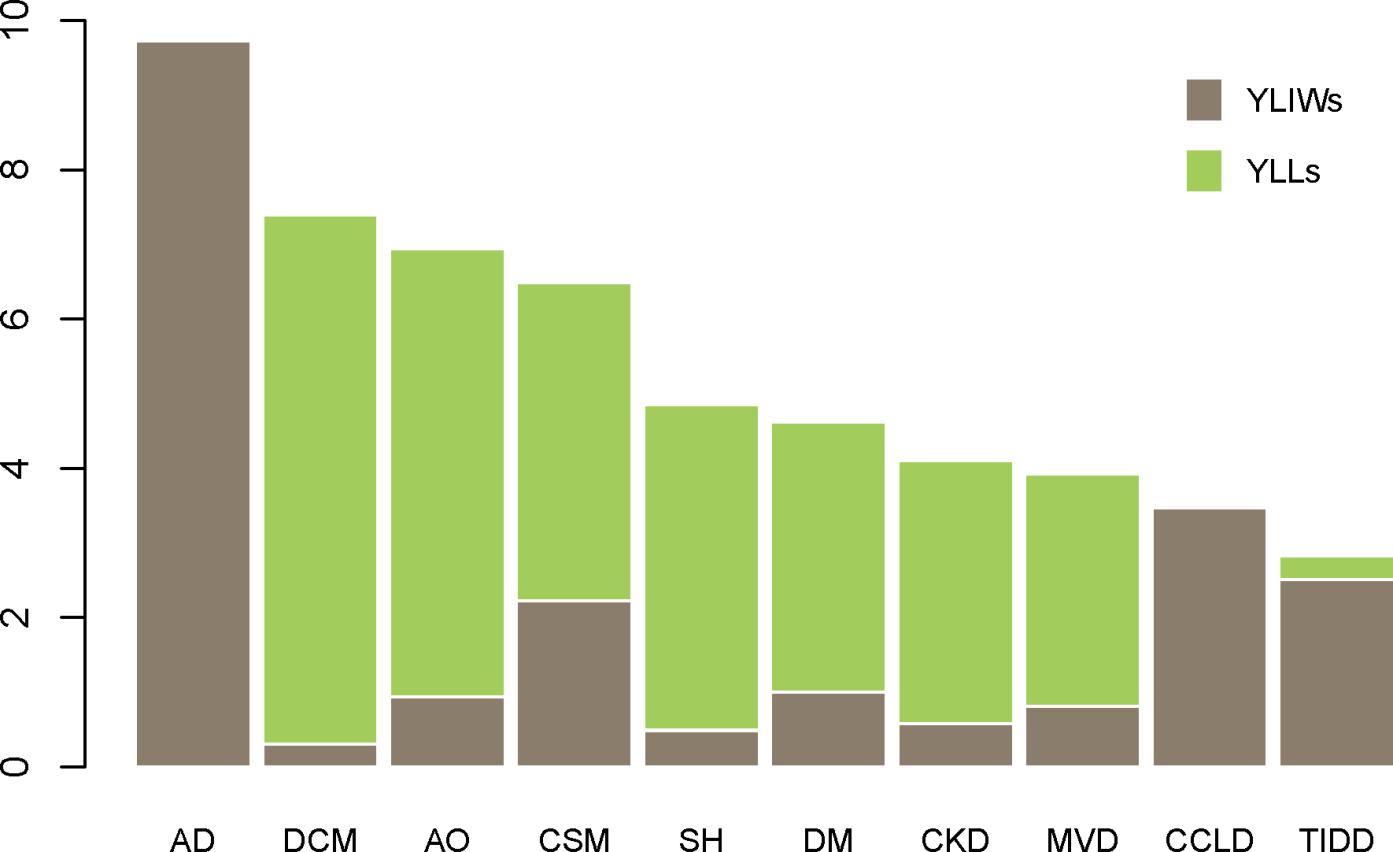
The composition of non-weighted welfare-adjusted life years for 10 canine diseases. The impaired welfare weights were anchored by time trade-off after excluding the impaired welfare weight of welfare impediment “respiratory distress” in the model building process. YLIWs: Years lived with impaired welfare; YLLs: years of life lost; AD: atopic dermatitis; DCM: dilated cardiomyopathy; AO: appendicular osteosarcoma; CSM: cervical spondylomyelopathy; SH: splenic haemangiosarcoma; DM: diabetes mellitus; CKD: chronic kidney disease; MVD: mitral valve disease; CCLD: cranial cruciate ligament disease; TIDD: thoracolumbar intervertebral disc disease.

YLIW, YLL, and WALY results using IWWs from each of the six loess regression models are shown in S5 Table. The WALY results using IWWs from the time trade-off method, after excluding the anchoring welfare impediment “respiratory distress” in the model building process, appear in Table 7 and Fig 2. The WALYs were very similar for both anchoring methods.

**Table 7:** Years lived with impaired welfare (YLIWs), non-weighted and weighted (with the impaired welfare weight of death) years of life lost (YLLs) due to premature death and non-weighted and weighted welfare-adjusted life years (WALY) for 10 canine diseases.

Variable	YLIW	Non-weighted YLLs	Non-weighted WALYs	Weighted YLLs	Weighted WALYs
Mitral valve disease	0.81 (0.14–1.70)	3.11 (1.10–7.08)	3.91 (1.80–7.67)	1.73 (0.60–3.98)	2.54 (1.21–4.61)
Dilated cardiomyopathy	0.31 (0.10–0.60)	7.09 (5.85–8.33)	7.40 (6.21–8.60)	3.96 (3.14–4.84)	4.27 (3.47–5.13)
Chronic kidney disease	0.58 (0.29–0.84)	3.52 (1.10–8.76)	4.11 (1.59–9.33)	1.97 (0.59–4.92)	2.55 (1.08–5.51)
Diabetes mellitus	1.00 (0.90–1.17)	3.62 (1.13–7.34)	4.62 (2.15–8.34)	2.02 (0.64–4.13)	3.02 (1.63–5.12)
Atopic dermatitis	9.73 (7.17–11.8)	0.00 (0.00–0.00)	9.73 (7.17–11.8)	0.00 (0.00–0.00)	9.73 (7.17–11.8)
Splenic haemangiosarcoma	0.49 (0.46–0.52)	4.36 (2.59–6.23)	4.85 (3.08–6.72)	2.43 (1.42–3.55)	2.93 (1.92–4.04)
Appendicular osteosarcoma	0.94 (0.67–1.30)	6.00 (2.90–9.90)	6.94 (3.83–10.84)	3.35 (1.60–5.59)	4.29 (2.52–6.55)
Cranial cruciate ligament disease	3.47 (2.39–4.58)	0.00 (0.00–0.00)	3.47 (2.39–4.58)	0.00 (0.00–0.00)	3.47 (2.39–4.58)
Thoracolumbar intervertebral disc disease	2.51 (1.37–3.51)	0.32 (0.16–0.45)	2.83 (1.54–3.94)	0.18 (0.09–0.26)	2.69 (1.47–3.75)
Cervical spondylomyelopathy	2.23 (1.67–3.17)	4.26 (2.28–6.63)	6.49 (4.55–8.76)	2.38 (1.26–3.76)	4.61 (3.40–6.00)

The impaired welfare weights were anchored by time trade-off (TTO) after excluding the impaired welfare weight of welfare impediment “respiratory distress” in the model building process.

Atopic dermatitis and thoracolumbar intervertebral disc disease had the highest and lowest WALYs at 9.73 (95% UI: 7.17–11.8) and 2.83 (95% UI: 1.54–3.94), respectively (Table 7). The WALYs for atopic dermatitis and cranial cruciate ligament disease were composed purely of YLIWs and the WALY for thoracolumbar intervertebral disc disease was composed mainly of YLIWs. In contrast, the WALYs of other diseases were derived predominantly from YLLs. As weighted YLLs were approximately 0.56 times of non-weighted YLLs, weighted WALYs were lower than non-weighted WALYs, apart from for atopic dermatitis and cranial cruciate ligament disease due to no YLLs.

## Discussion

To the authors’ knowledge, WALY is the first metric to estimate the total disease impact of both welfare compromise and premature death in animals. This study created the WALY metric and IWW generation as an adaptation of the DALY metric and disability weight index used in human medicine, respectively, and generated WALYs for ten canine diseases and estimated IWWs for 35 associated welfare impediments. These approaches revealed recommendations that are expected to improve future applications of the methodology.

There are some important differences between the DALY and WALY assessments. Firstly, DALY focuses more on population disease burden whereas WALY puts more emphasis on individual animal welfare. That said, population-level WALYs can be calculated by multiplying the number of cases in a certain population. Secondly, in WALY, we intended to quantify “impaired welfare” instead of “impaired health”, as is used in DALYs. There is a notable difference in the definition of health between humans and animals. For human health, the WHO definition is “a state of complete physical, mental and social well-being and not merely the absence of disease or infirmity” [54]. Yet health in veterinary contexts is not well characterised and generally focuses on physical functionality [55]. The health of animals is considered a crucial component of welfare and so is not commonly considered the same as well-being (i.e., welfare), as it is in humans [56]. Therefore, in the context of non-human animals, “welfare” captures more negative impacts from diseases or other causes than “health” alone does.

As we tried to quantify the impairment of welfare instead of the impairment of health, one philosophical question arose: can “death” be a welfare impediment and where should it be located on the spectrum of welfare impediments? In DALYs, death is not a health state as it represents a total loss of health. However, there are situations when people consider death is better than being alive and terminate their life via voluntary euthanasia [57]. In the EuroQol- 5 dimensions questionnaire, an instrument widely used to evaluate health-related quality of life in humans, “health state(s) worse than death” is a complex, and largely unresolved, issue [27, 58]. Therefore, in the current study, death was included as a welfare impediment such that the current weighted YLLs and weighted WALYs incorporate the IWW of death. Interestingly, death emerged with an IWW of approximately 0.56. As an IWW of 0 is the optimal welfare imaginable and 1 is the worst welfare that one can imagine, a value close to 0.5 might reflect the notion that death is a neutral welfare impediment where positive and negative experiences are equally balanced (both zero in the case of death). However, because death is often seen as more of an ethical than a welfare issue [59], we estimated weighted YLLs and WALYs only for the purpose of encouraging discussion.

There are two distinct types of health/welfare impediments that can be used in DALY/WALY, namely cause-specific and generic. Cause-specific welfare impediments mean that each cause is one welfare impediment, while generic welfare impediments, such as the welfare impediments in the current study, are not specific to a single cause and can result from several causes. Both cause-specific and generic welfare impediments have their advantages. WALY calculations using cause-specific welfare impediments require less input because each disease has one only welfare impediment. Also, in the companion animal field, corresponding epidemiological frequency and/or duration data for diseases are more abundant and detailed than signs of diseases. However, there are two main concerns associated with the use of cause-specific welfare impediments. The first is the putative influence of the disease label (i.e., the name of the disease) on people’s responses. Stouthard et al. (2000) reported an instance when, for two diseases with similar health profiles, people judged the severity of the diseases differently upon seeing their disease labels [60]. This indicates that disease labels provide information that does not necessarily reflect the true state of the patient’s health but can affect health evaluation. Secondly, IWWs of the cause-specific welfare impediments may need to be measured repeatedly because the welfare compromises caused by these diseases might lessen with advances in medicine over time. In contrast, WALY calculation using the IWWs of generic welfare impediments requires only an update of the duration and prevalence of the welfare impediments. Unlike cause-specific welfare impediments where the scale of the welfare impediments is consistent, the scale of generic welfare impediments can vary because some, such as “severe heart failure (with treatment)”, involve multiple signs, whereas others are just a single sign. The same proviso applies to the health states in the GBD 2010 and 2013, so it does not concern us excessively, especially because the evaluation standards for welfare compromise in the current study were consistent across all the welfare impediments. Generation of a single weight for a cause-specific or generic welfare impediment can over-simplify the severity of the welfare impediment. Whether or not the IWW generated in the current report reflects the actual welfare impact partially relies on the acumen of the researchers and the experts consulted.

The current study adopted the methodology used in the generation of disability weights for health states in GBD 2010 and 2013 and it elicited IWWs based on data from paired comparison questions along with anchoring methods, visual analogue scale and time trade-offs. It is prudent to note that the WALY measures animal welfare as perceived by humans (in this case veterinarians), and not as perceived by the animals, i.e., dogs in the current study. In the current study, the visual analogue scale and time trade-off were selected as the anchoring methods for several reasons. A visual analogue scale is often used in disability weight generation in DALY [61] and is relatively straightforward, compared with trade-off methods. Although it has been reported that visual analogue scales generate higher disability weights than other methods, including time trade-off, and relatively high disability weights for mild conditions [62], this outcome was not observed in the current study when the IWWs anchored by the visual analogue scale were compared with those anchored by time trade-off. Trade-off methods share a degree of cognitive complexity which, in the current study, was confirmed firstly by the feedback from some participants about the time trade-off module and, secondly, by the wider range and variance across the five IWWs elicited by time trade-off than those by visual analogue scale. However, the IWWs rescaled by time trade-off and those rescaled by visual analogue scale were fairly similar. A possible inherent problem with time trade-off may be that people value future life at a lower rate than current life [19]. However, this may be less of a concern in the current study than in studies of human health states that have used time trade-off to generate disability weights because people are less likely to put themselves fully into the dogs’ position. One limitation emerged with the design of the time trade-off questions in the current study: Although trade-off questions were hypothetical, it was virtually impossible to imagine dogs with some of the welfare impediments, such as “respiratory distress”, in the time trade-off module having to live for 10 years. We suggest future studies consider using the population health equivalence, a different trade-off anchoring method that asks participants to judge the overall population health benefits produced by two different preventative programs [23] or reducing the putative length of time if using time trade-off.

Some results of the current study ran counter to our expectations. The ranking of the IWWs for “respiratory distress” and “cancer: lung metastasis with/without having metastasis in other parts of the body” differed between the paired comparison results and results from both visual analogue scale and time trade-off modules. Logical inconsistency, that is, the situation when worse welfare/health states are valued as less severe than better welfare/health states, has also occurred in the EuroQol- 5 dimensions questionnaire. Such logical inconsistency has been observed primarily among male, older, less-educated and religious participants in studies of human health states conducted in Spain, Netherland and China [63–65]. Moreover, when an interview is needed for the valuation, the risk of logical inconsistency is also associated with interviewers’ performance and the interview process [65]. We judged that visual analogue scale and time trade-off reflected the true rankings better than paired comparison. On the visual analogue scale, the five welfare impediments were compared directly, and, in time trade-off, participants were likely to compare the five welfare impediments when contemplating the period to be traded-off. In contrast, the variety of comparisons of welfare impediments in the paired comparison module is much greater than the comparisons in the visual analogue scale and the time trade-off modules, and is comparatively indirect. Additionally, as with many questionnaires, there may be a so-called learning curve for participants as they engaged with the various stages of the current survey. Because the participants started with paired comparison questions, followed by the visual analogue and time trade-off, the pattern of comparisons would have been more consistent later than earlier. Similarly (and surprisingly), the IWW was higher in “overweight” than in “obesity,” and higher in “spinal hyperesthesia: mild” than in “spinal hyperesthesia: severe.” As paired comparison methodology has been used to generate disability weights in various studies [23, 66, 67], and we were content that the severity of any welfare compromise was discernible from the lay descriptions when compared directly, the limited combinations of paired comparison questions used in the questionnaires are likely to be responsible for these unexpected results. In GBD, an algorithm was used for the random selection of health states for paired comparison questions, so combinations of paired comparison questions in that device were unlimited [23]. In contrast, the current study offered limited combinations of the paired comparison questions which might inadvertently impose a pattern of responses resulting in a systemic error. We suggest an algorithm allowing random selection of paired comparison questions should be used in the future.

Because of the unattainability of verbal communication between human and non-human animals, animal welfare evaluations are often vulnerable to a certain level of subjectivity on the part of human evaluators [68]. IWW evaluation is not an exception. Many factors may influence the welfare evaluation, including the attitudes towards animal welfare and the ability to judge the degree of welfare compromise accurately. Although many of those factors were explored in the questionnaire, the current sample size was not generated specifically to identify the statistical difference in IWWs between different characteristics of the respondents. Nevertheless, some trends were observed. For example, anchoring IWW values were higher in females than males. This finding is unsurprising, given that gender is often associated with attitudes towards animals and animal welfare [24, 25] and females often show more empathy to animals [25, 69]. Also, veterinarians who were younger or graduated recently perceived higher IWWs for the five anchoring welfare impediments. This might relate directly to the length of their time in practice which may inadvertently have a desensitising effect as less concern for animal sentience has been shown among students in the higher years of veterinary study [24]. It is recognised that veterinarians continuously encounter ethical conflicts and moral distress during practice [70]. So, counter-anthropomorphising, a process of assigning inanimate qualities to living things, may distance them from those challenges and also desensitise them to compromised welfare in animals around them [71]. On the other hand, more emphasis on animal welfare education in veterinary schools in recent years may have given recent graduates more awareness of animal welfare and animal sentience than the older graduates, allowing them to make a more knowledge-based evaluation [72, 73]. Moreover, as the veterinary profession is feminising [74], there is probably an ongoing interaction of gender and age. Veterinarians working with livestock rated the five anchoring welfare impediments as less severe than those not working with livestock. Farming has been found to be associated with instrumental attitudes towards animals [25, 26]; an attitude that may have carried over to the dogs in the current hypothetical cases. We acknowledge a likely interaction between working with livestock and gender because males in our study population were 1.3 times more likely to work with livestock than females were.

Another factor potentially affecting the current values of IWWs is the source of the participants [75]. There are debates about the most suitable population from which to recruit participants to elicit disability weights [76]. Although an increasing number of studies that elicited the disability weights for DALYs have been recruited from the general public [23, 66, 67], early studies often targeted health professionals’ opinions [20, 77, 78]. This latter practice was criticised because health professionals are likely to link the lay descriptions to specific disease labels (whose inadvertent influence have been discussed above) and unintentionally to take into account comorbidities of the diseases, resulting in an overestimation of disability weights [23]. Veterinarians were targeted for the current study, instead of lay dog owners, for two reasons. Firstly, we expected lay dog owners to be equipped with less knowledge of animal welfare than veterinarians. Secondly, it is more likely that lay dog owners would evaluate dog welfare anthropomorphically. In the current study, more than 90% of the participants grew up with dogs or had owned dogs, meaning that, beyond their clinical insights, they also understood dog welfare from an owner’s perspective. However, it would be interesting to use the current tool to elicit IWWs from lay dog owners and to compare them with those from veterinarians. Paired comparison questions could be easily used in such a study, because they have been shown to be comprehensible by people with various levels of education, in contrast to commonly used trade-off methods [23].

There are some more limitations of the current study that we wish to acknowledge. Firstly, the sources of the published articles that were used to obtain the epidemiological information for WALY calculation varied. As shown in Table 1, some articles were fairly old and so might not depict the current situation, and some of the prevalence and duration of the welfare impediments in the same disease were necessarily sourced from different articles. Additionally, it is likely that the epidemiological information reported in some of these articles was not representative of the general dog population as most of the articles used data from premium veterinary practices, particularly teaching hospitals. Ideally, all the epidemiological information should come from the same population of dogs, temporally and spatially. Data from well-constructed companion animal databases, such as VetCompass in the UK [79] and Australia [80] and Agria insurance company in Sweden [81], would be needed to achieve such goals. Secondly, as the evaluation of the IWWs was based on the impact on dogs’ biological functioning evaluated by veterinarians instead of their emotive states, dogs might not necessarily prefer a welfare impediment with lower IWW. Thirdly, the prospects of comorbidity were not embraced in this study. These merit consideration. On the one hand, it is likely that the total welfare compromise caused by having both welfare impediment “A” and welfare impediment “B” concurrently may not amount to a simple summation of their welfare compromise. On the other hand, we did not embed methods to account for conflated welfare impediments in animals with more than one disease that have the same welfare impediments. In this case, the YLIWs for affected animals would be overestimated. The sample size needs to be larger to account for potential confounders, such as gender and age, and to allow the consideration of the variability of the anchoring IWWs in the probit model. Fourthly, the mortality caused by euthanasia in atopic dermatitis and cranial cruciate ligament disease was not factored into the current WALY calculations due to the unavailability of the relevant information in the literature. Lastly, although the methods used to elicit IWWs have been validated in GBD 2010 and 2013 [23, 66], validation of the current approach to (companion) animals is needed.

The WALY approach has shown promise in the current pilot and may have extensive applications. Firstly, the methodology allows welfare compromise and total disease impact of all sorts of diseases to be quantified and compared. It has the potential to inform prioritisation of research into prevention of and interventions for the diseases that have more adverse effects. Also, analyses of the cost-effectiveness of disease prevention merit exploration. Secondly, WALYs can also be estimated for populations. WALYs for diseases can be calculated for different sex, breed, age groups and geographical regions and then compared. This is particularly useful for determining the total impact of diseases in different breeds of dogs [18]. A quantitative exploration of breeds with poor welfare as a result of greater disease burden can be accomplished by comparing the summated WALY values of common diseases for different breeds of dogs. However, even for an identical welfare impediment, the welfare compromises can vary among breeds and, especially among different sizes and shapes of dogs. For instance, anecdotally, the welfare of paralysed large dogs is much worse than paralysed small dogs, and they require more care from humans than paralysed small dogs do. This may need to be factored into further considerations of IWW. Thirdly, the WALY would allow monitoring of changes in the welfare and disease impact of a given population over time. Fourthly, WALYs can be calculated not only for the impact of diseases but also of other welfare issues, such as long-term confinement, training interventions or undesirable behaviours. Lastly, although we demonstrated the WALY framework with diseases in dogs, it can also be adapted for use in other companion and production animals. However, if using WALY metric for other species of animal, the unit of time may need to be changed from year to a unit of time more suitable for that species, depending on the expected lifespan of the species and the usage of the animal in human contexts. The expected lifespan in production animals would then be the time from birth to slaughter or culling. Instead of units of time, using the proportion of life affected will enable the comparison across species.

## Conclusion

This study developed the WALY metric by adapting the DALY approach from the GBD studies. The approach was implemented for 10 common canine diseases that not only enabled us to estimate IWWs and WALYs but also allowed the identification of potential issues that could affect this calculation and could be improved upon in the future.

The WALY could become a powerful metric to summarise welfare compromise and disease impact of individual diseases in companion and production animals. Future potential applications of the WALY are extensive and may include prioritisation of disorders for eradication and control, quantification of population welfare and longitudinal surveillance of animal welfare.

## Supporting information

S1 File A version of the questionnaire for eliciting impaired welfare weight. (PPTX)

S1 Table Lay descriptions and welfare compromises classified by Five Domain of 35 welfare impediments. (DOCX)

S2 Table An excerpt of the paired comparison dataset. (XLSX)

S3 Table Demographics of participants who completed questionnaires for IWW generation. (XLSX)

S4 Table Impaired welfare weight results elicited by the six loess regression models in the study. (XLSX)

S5 Table Years lived with impaired welfare, years of life lost, and welfare-adjusted life year results using impaired welfare weights elicited by each of the six loess regression models. (XLSX)
