# Supplementary material for: Welfare-Adjusted Life Years (WALY): A novel metric of animal welfare that combines the impacts of impaired welfare and abbreviated lifespan
Source: PLoS One. 2018 Sep 12;13(9):e0202580. doi: 10.1371/journal.pone.0202580 (PMC6135394; doi:10.1371/journal.pone.0202580)
Supplement: S1 File — (PPTX) [file pone.0202580.s001.pptx]

## Slide 1
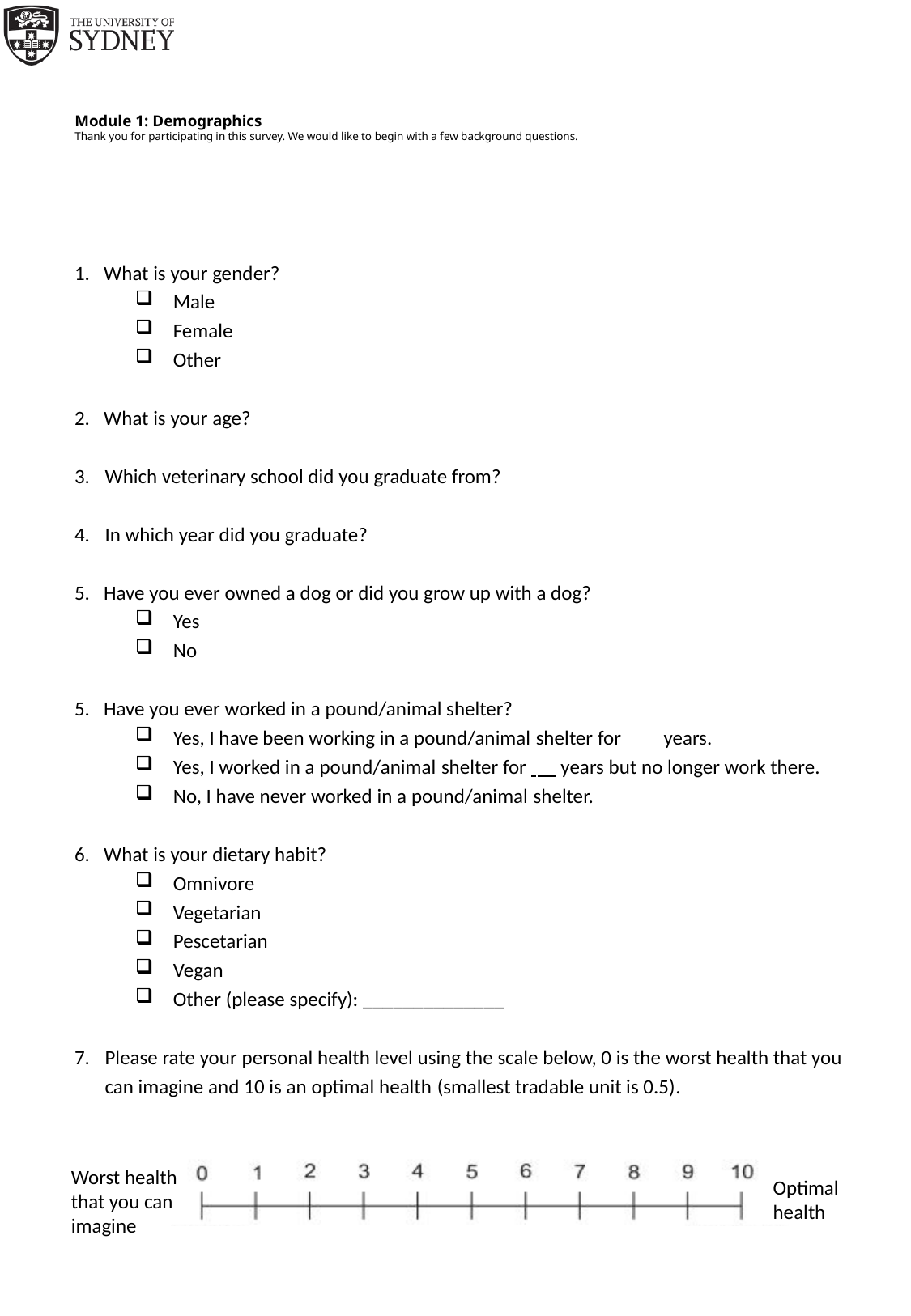

# Module 1: Demographics Thank you for participating in this survey. We would like to begin with a few background questions.
1. What is your gender?
Male
Female
Other
2. What is your age?
Which veterinary school did you graduate from?
In which year did you graduate?
5. Have you ever owned a dog or did you grow up with a dog?
Yes
No
5. Have you ever worked in a pound/animal shelter?
Yes, I have been working in a pound/animal shelter for 	 years.
Yes, I worked in a pound/animal shelter for 	 years but no longer work there.
No, I have never worked in a pound/animal shelter.
6. What is your dietary habit?
Omnivore
Vegetarian
Pescetarian
Vegan
Other (please specify): ______________
Please rate your personal health level using the scale below, 0 is the worst health that you can imagine and 10 is an optimal health (smallest tradable unit is 0.5).
Worst health that you can imagine
Optimal health

## Slide 2
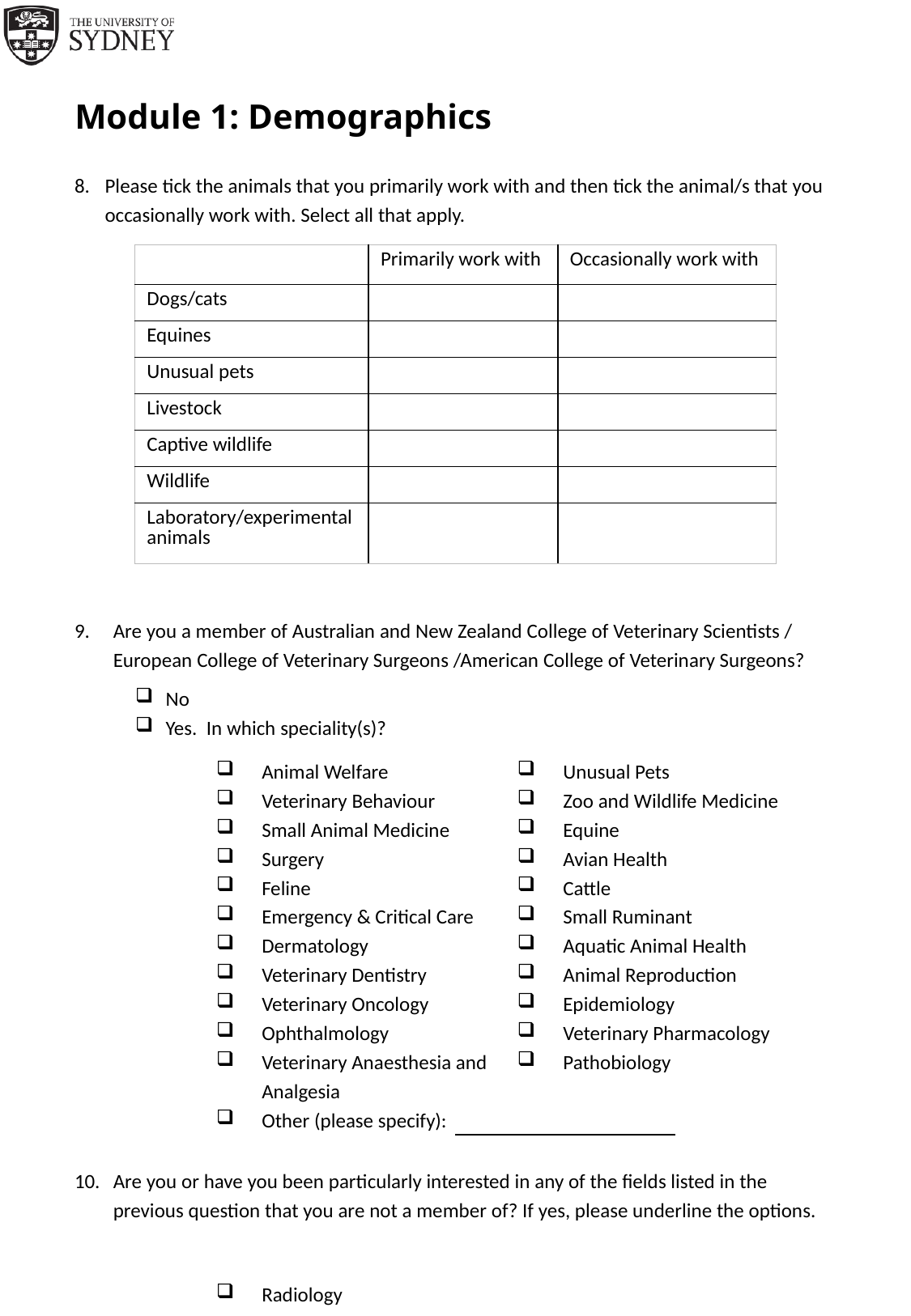

# Module 1: Demographics
Please tick the animals that you primarily work with and then tick the animal/s that you occasionally work with. Select all that apply.
Are you a member of Australian and New Zealand College of Veterinary Scientists / European College of Veterinary Surgeons /American College of Veterinary Surgeons?
No
Yes. In which speciality(s)?
Are you or have you been particularly interested in any of the fields listed in the previous question that you are not a member of? If yes, please underline the options.
| | Primarily work with | Occasionally work with |
| --- | --- | --- |
| Dogs/cats | | |
| Equines | | |
| Unusual pets | | |
| Livestock | | |
| Captive wildlife | | |
| Wildlife | | |
| Laboratory/experimental animals | | |
Animal Welfare
Veterinary Behaviour
Small Animal Medicine
Surgery
Feline
Emergency & Critical Care
Dermatology
Veterinary Dentistry
Veterinary Oncology
Ophthalmology
Veterinary Anaesthesia and Analgesia
Other (please specify):
Radiology
Unusual Pets
Zoo and Wildlife Medicine
Equine
Avian Health
Cattle
Small Ruminant
Aquatic Animal Health
Animal Reproduction
Epidemiology
Veterinary Pharmacology
Pathobiology

## Slide 3
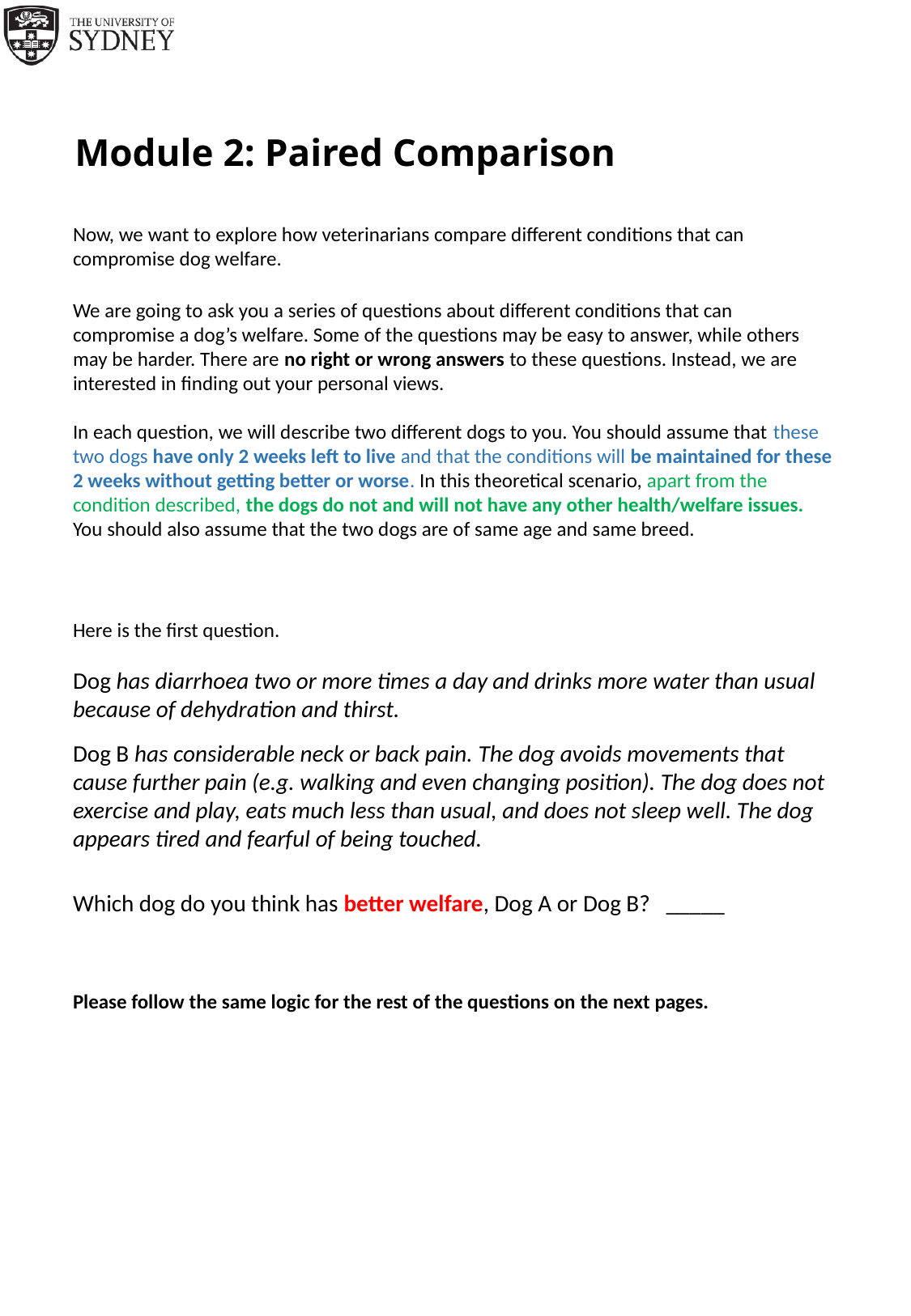

# Module 2: Paired Comparison
Now, we want to explore how veterinarians compare different conditions that can compromise dog welfare.
We are going to ask you a series of questions about different conditions that can compromise a dog’s welfare. Some of the questions may be easy to answer, while others may be harder. There are no right or wrong answers to these questions. Instead, we are interested in finding out your personal views.
In each question, we will describe two different dogs to you. You should assume that these two dogs have only 2 weeks left to live and that the conditions will be maintained for these 2 weeks without getting better or worse. In this theoretical scenario, apart from the condition described, the dogs do not and will not have any other health/welfare issues. You should also assume that the two dogs are of same age and same breed.
Here is the first question.
Dog has diarrhoea two or more times a day and drinks more water than usual because of dehydration and thirst.
Dog B has considerable neck or back pain. The dog avoids movements that cause further pain (e.g. walking and even changing position). The dog does not exercise and play, eats much less than usual, and does not sleep well. The dog appears tired and fearful of being touched.
Which dog do you think has better welfare, Dog A or Dog B? _____
Please follow the same logic for the rest of the questions on the next pages.

## Slide 4
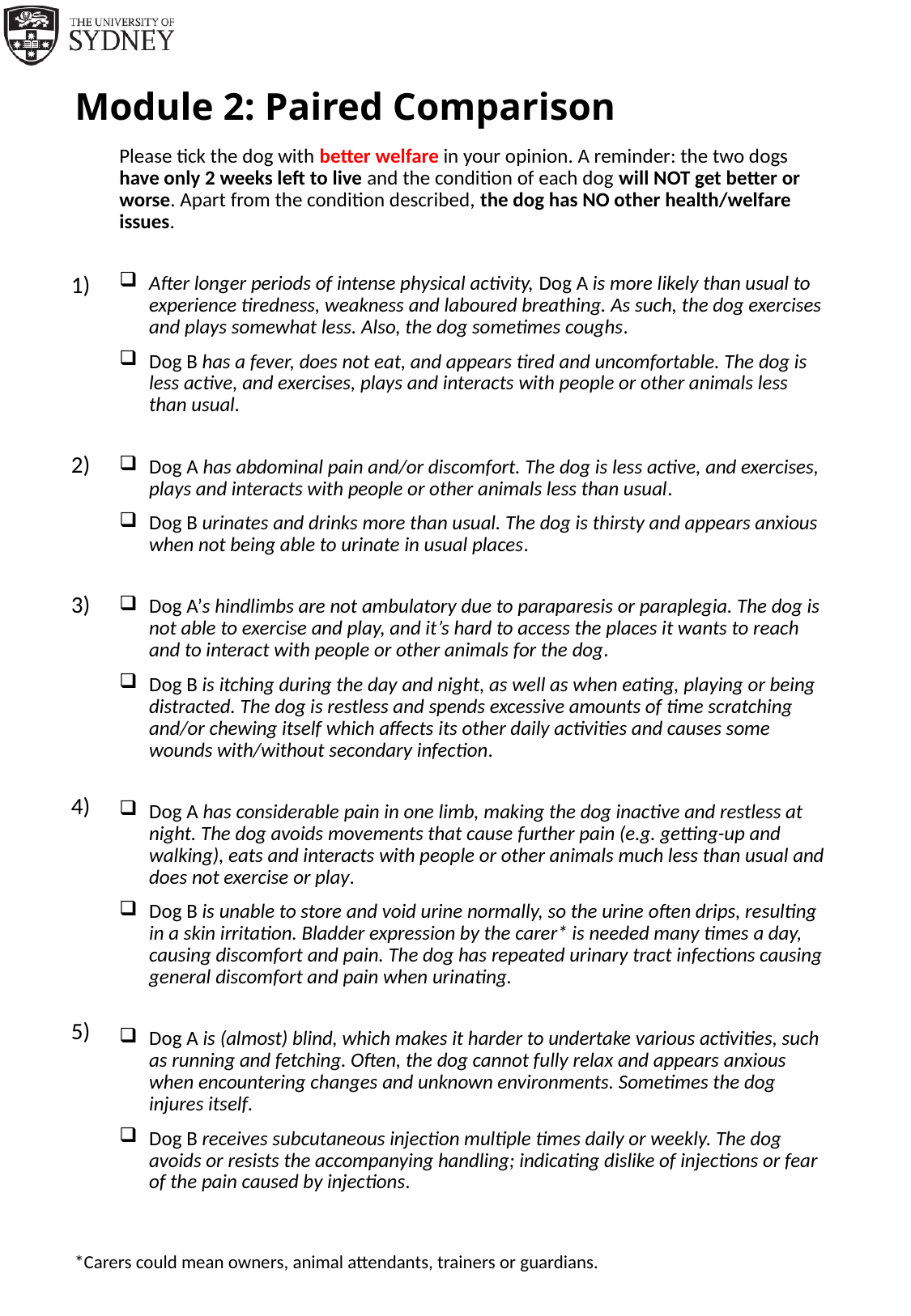

# Module 2: Paired Comparison
Please tick the dog with better welfare in your opinion. A reminder: the two dogs have only 2 weeks left to live and the condition of each dog will NOT get better or worse. Apart from the condition described, the dog has NO other health/welfare issues.
After longer periods of intense physical activity, Dog A is more likely than usual to experience tiredness, weakness and laboured breathing. As such, the dog exercises and plays somewhat less. Also, the dog sometimes coughs.
Dog B has a fever, does not eat, and appears tired and uncomfortable. The dog is less active, and exercises, plays and interacts with people or other animals less than usual.
Dog A has abdominal pain and/or discomfort. The dog is less active, and exercises, plays and interacts with people or other animals less than usual.
Dog B urinates and drinks more than usual. The dog is thirsty and appears anxious when not being able to urinate in usual places.
Dog A’s hindlimbs are not ambulatory due to paraparesis or paraplegia. The dog is not able to exercise and play, and it’s hard to access the places it wants to reach and to interact with people or other animals for the dog.
Dog B is itching during the day and night, as well as when eating, playing or being distracted. The dog is restless and spends excessive amounts of time scratching and/or chewing itself which affects its other daily activities and causes some wounds with/without secondary infection.
Dog A has considerable pain in one limb, making the dog inactive and restless at night. The dog avoids movements that cause further pain (e.g. getting-up and walking), eats and interacts with people or other animals much less than usual and does not exercise or play.
Dog B is unable to store and void urine normally, so the urine often drips, resulting in a skin irritation. Bladder expression by the carer* is needed many times a day, causing discomfort and pain. The dog has repeated urinary tract infections causing general discomfort and pain when urinating.
Dog A is (almost) blind, which makes it harder to undertake various activities, such as running and fetching. Often, the dog cannot fully relax and appears anxious when encountering changes and unknown environments. Sometimes the dog injures itself.
Dog B receives subcutaneous injection multiple times daily or weekly. The dog avoids or resists the accompanying handling; indicating dislike of injections or fear of the pain caused by injections.
*Carers could mean owners, animal attendants, trainers or guardians.

## Slide 5
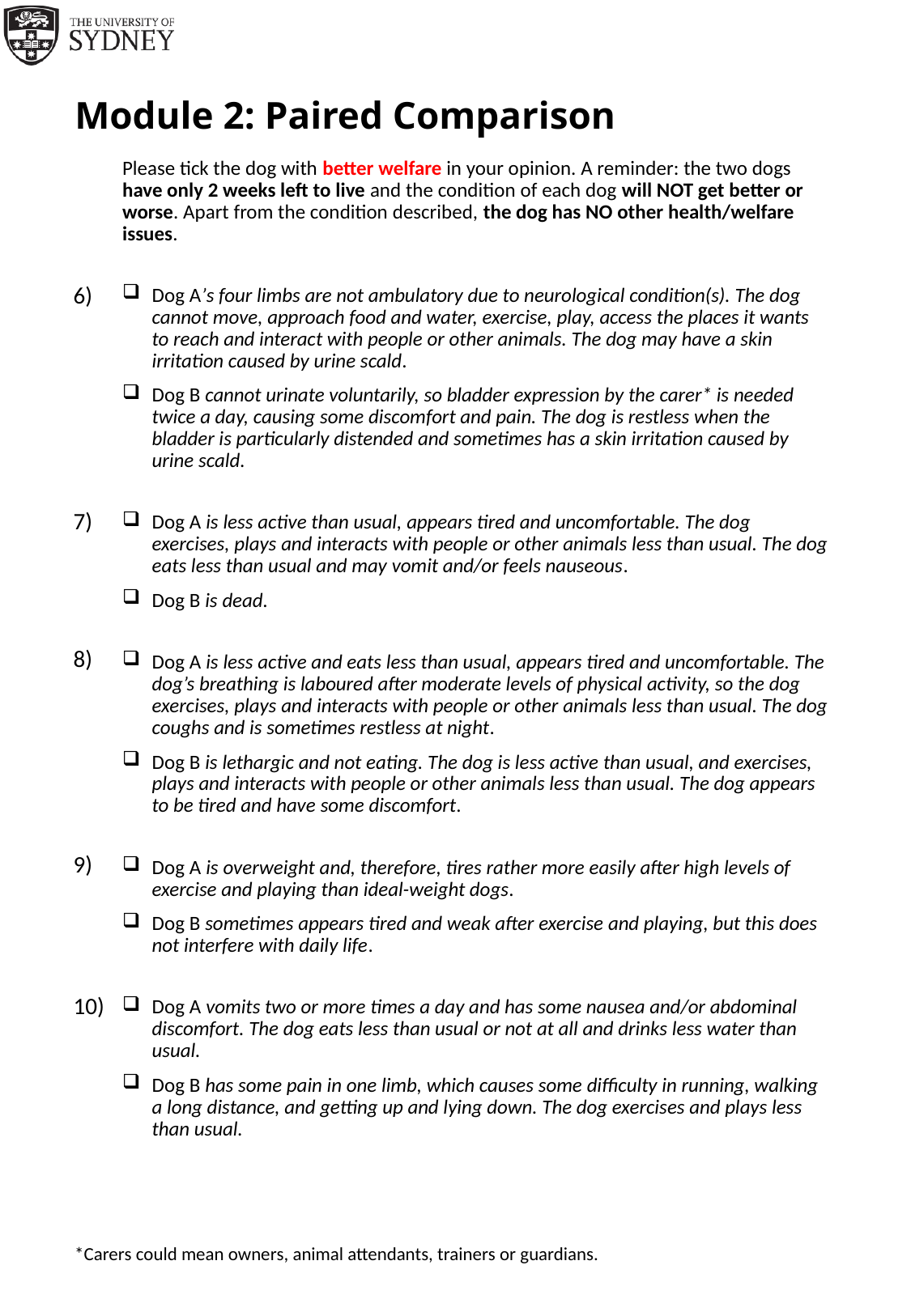

# Module 2: Paired Comparison
Please tick the dog with better welfare in your opinion. A reminder: the two dogs have only 2 weeks left to live and the condition of each dog will NOT get better or worse. Apart from the condition described, the dog has NO other health/welfare issues.
Dog A’s four limbs are not ambulatory due to neurological condition(s). The dog cannot move, approach food and water, exercise, play, access the places it wants to reach and interact with people or other animals. The dog may have a skin irritation caused by urine scald.
Dog B cannot urinate voluntarily, so bladder expression by the carer* is needed twice a day, causing some discomfort and pain. The dog is restless when the bladder is particularly distended and sometimes has a skin irritation caused by urine scald.
Dog A is less active than usual, appears tired and uncomfortable. The dog exercises, plays and interacts with people or other animals less than usual. The dog eats less than usual and may vomit and/or feels nauseous.
Dog B is dead.
Dog A is less active and eats less than usual, appears tired and uncomfortable. The dog’s breathing is laboured after moderate levels of physical activity, so the dog exercises, plays and interacts with people or other animals less than usual. The dog coughs and is sometimes restless at night.
Dog B is lethargic and not eating. The dog is less active than usual, and exercises, plays and interacts with people or other animals less than usual. The dog appears to be tired and have some discomfort.
Dog A is overweight and, therefore, tires rather more easily after high levels of exercise and playing than ideal-weight dogs.
Dog B sometimes appears tired and weak after exercise and playing, but this does not interfere with daily life.
Dog A vomits two or more times a day and has some nausea and/or abdominal discomfort. The dog eats less than usual or not at all and drinks less water than usual.
Dog B has some pain in one limb, which causes some difficulty in running, walking a long distance, and getting up and lying down. The dog exercises and plays less than usual.
*Carers could mean owners, animal attendants, trainers or guardians.

## Slide 6
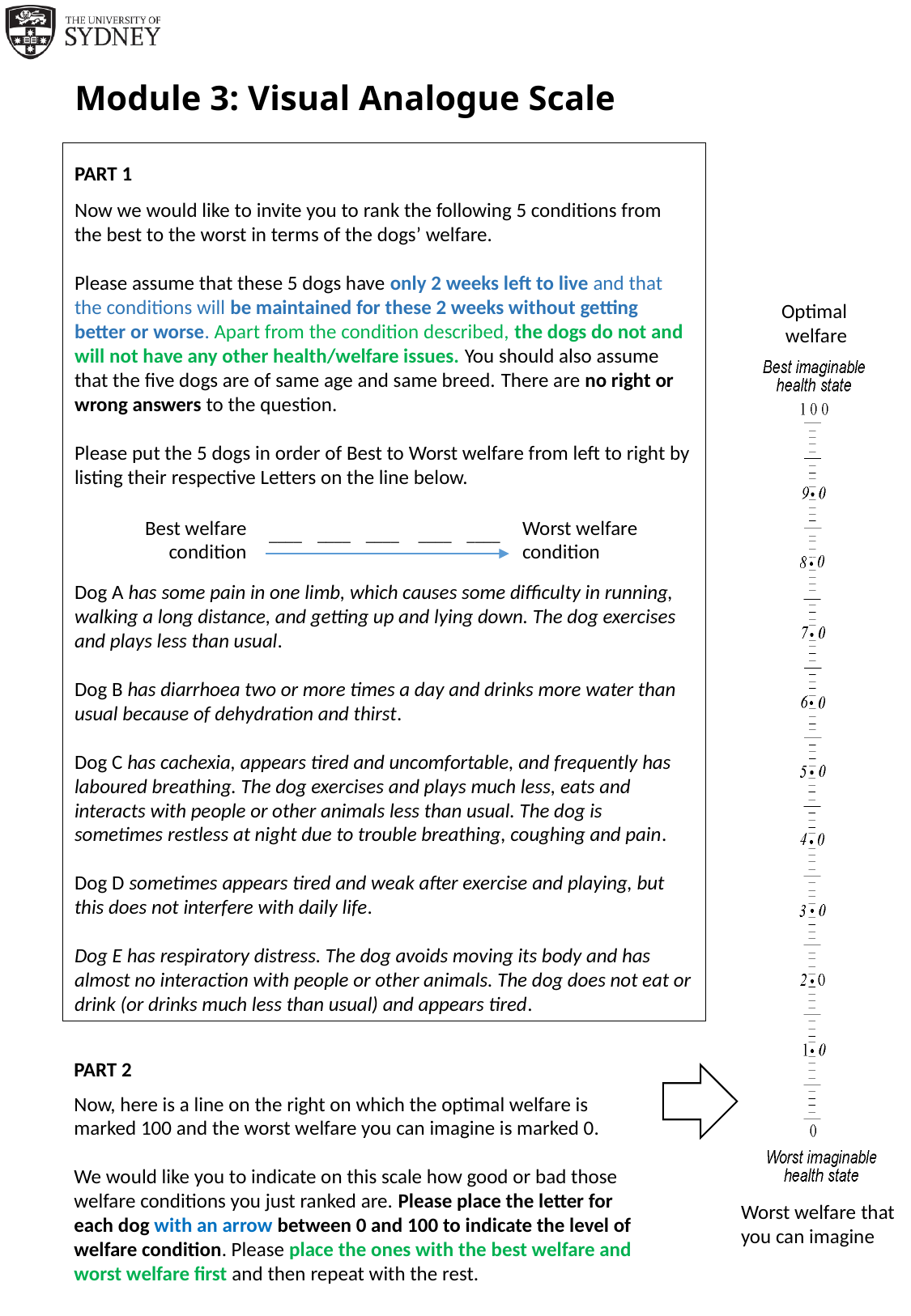

# Module 3: Visual Analogue Scale
PART 1
Now we would like to invite you to rank the following 5 conditions from the best to the worst in terms of the dogs’ welfare.
Please assume that these 5 dogs have only 2 weeks left to live and that the conditions will be maintained for these 2 weeks without getting better or worse. Apart from the condition described, the dogs do not and will not have any other health/welfare issues. You should also assume that the five dogs are of same age and same breed. There are no right or wrong answers to the question.
Please put the 5 dogs in order of Best to Worst welfare from left to right by listing their respective Letters on the line below.
 	 ____ ____ ____ ____ ____
Dog A has some pain in one limb, which causes some difficulty in running, walking a long distance, and getting up and lying down. The dog exercises and plays less than usual.
Dog B has diarrhoea two or more times a day and drinks more water than usual because of dehydration and thirst.
Dog C has cachexia, appears tired and uncomfortable, and frequently has laboured breathing. The dog exercises and plays much less, eats and interacts with people or other animals less than usual. The dog is sometimes restless at night due to trouble breathing, coughing and pain.
Dog D sometimes appears tired and weak after exercise and playing, but this does not interfere with daily life.
Dog E has respiratory distress. The dog avoids moving its body and has almost no interaction with people or other animals. The dog does not eat or drink (or drinks much less than usual) and appears tired.
Optimal welfare
Worst welfare that you can imagine
Best welfare condition
Worst welfare condition
PART 2
Now, here is a line on the right on which the optimal welfare is marked 100 and the worst welfare you can imagine is marked 0.
We would like you to indicate on this scale how good or bad those welfare conditions you just ranked are. Please place the letter for each dog with an arrow between 0 and 100 to indicate the level of welfare condition. Please place the ones with the best welfare and worst welfare first and then repeat with the rest.

## Slide 7
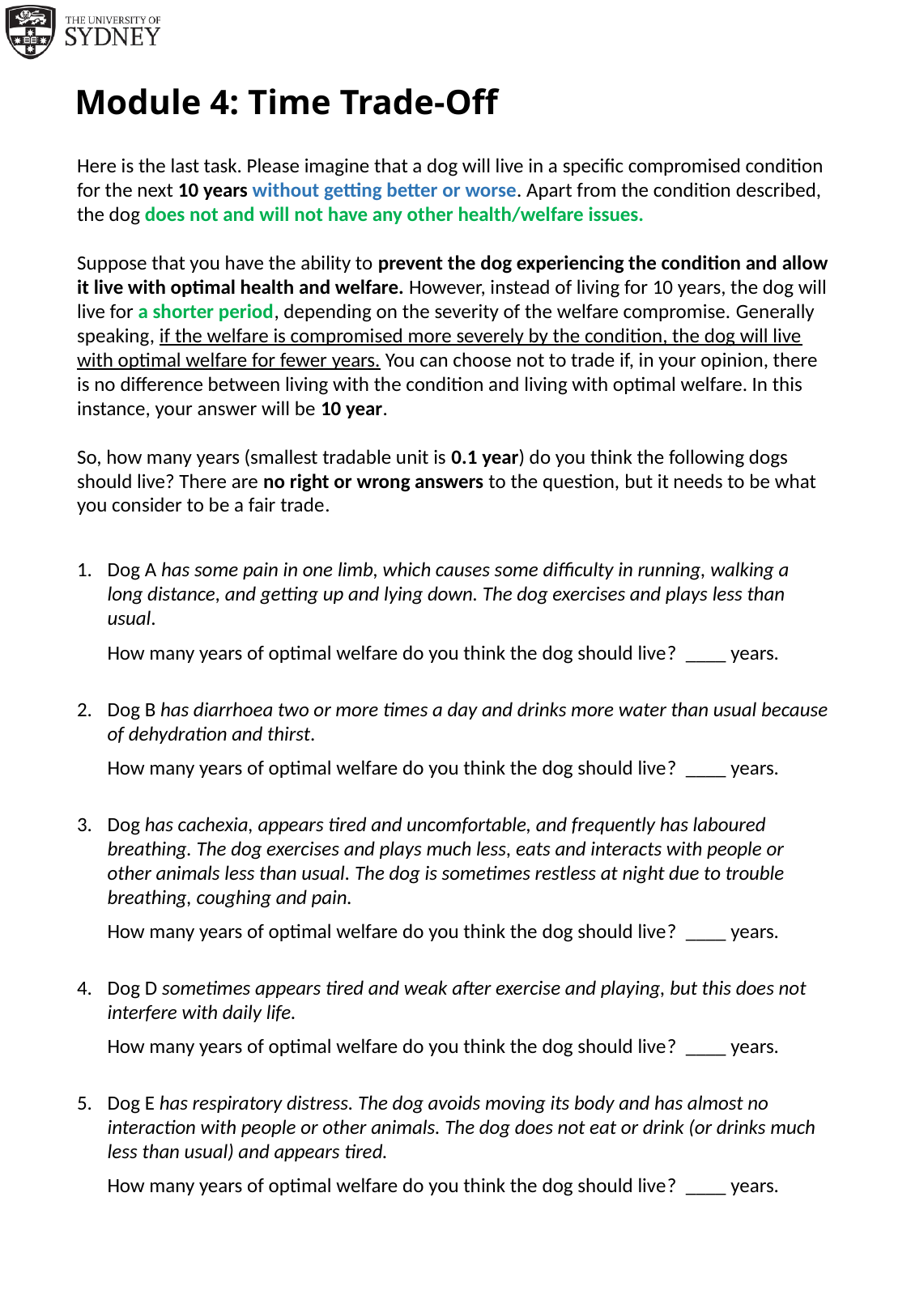

Module 4: Time Trade-Off
Here is the last task. Please imagine that a dog will live in a specific compromised condition for the next 10 years without getting better or worse. Apart from the condition described, the dog does not and will not have any other health/welfare issues.
Suppose that you have the ability to prevent the dog experiencing the condition and allow it live with optimal health and welfare. However, instead of living for 10 years, the dog will live for a shorter period, depending on the severity of the welfare compromise. Generally speaking, if the welfare is compromised more severely by the condition, the dog will live with optimal welfare for fewer years. You can choose not to trade if, in your opinion, there is no difference between living with the condition and living with optimal welfare. In this instance, your answer will be 10 year.
So, how many years (smallest tradable unit is 0.1 year) do you think the following dogs should live? There are no right or wrong answers to the question, but it needs to be what you consider to be a fair trade.
Dog A has some pain in one limb, which causes some difficulty in running, walking a long distance, and getting up and lying down. The dog exercises and plays less than usual.
How many years of optimal welfare do you think the dog should live? ____ years.
Dog B has diarrhoea two or more times a day and drinks more water than usual because of dehydration and thirst.
How many years of optimal welfare do you think the dog should live? ____ years.
Dog has cachexia, appears tired and uncomfortable, and frequently has laboured breathing. The dog exercises and plays much less, eats and interacts with people or other animals less than usual. The dog is sometimes restless at night due to trouble breathing, coughing and pain.
How many years of optimal welfare do you think the dog should live? ____ years.
Dog D sometimes appears tired and weak after exercise and playing, but this does not interfere with daily life.
How many years of optimal welfare do you think the dog should live? ____ years.
Dog E has respiratory distress. The dog avoids moving its body and has almost no interaction with people or other animals. The dog does not eat or drink (or drinks much less than usual) and appears tired.
How many years of optimal welfare do you think the dog should live? ____ years.

## Slide 8
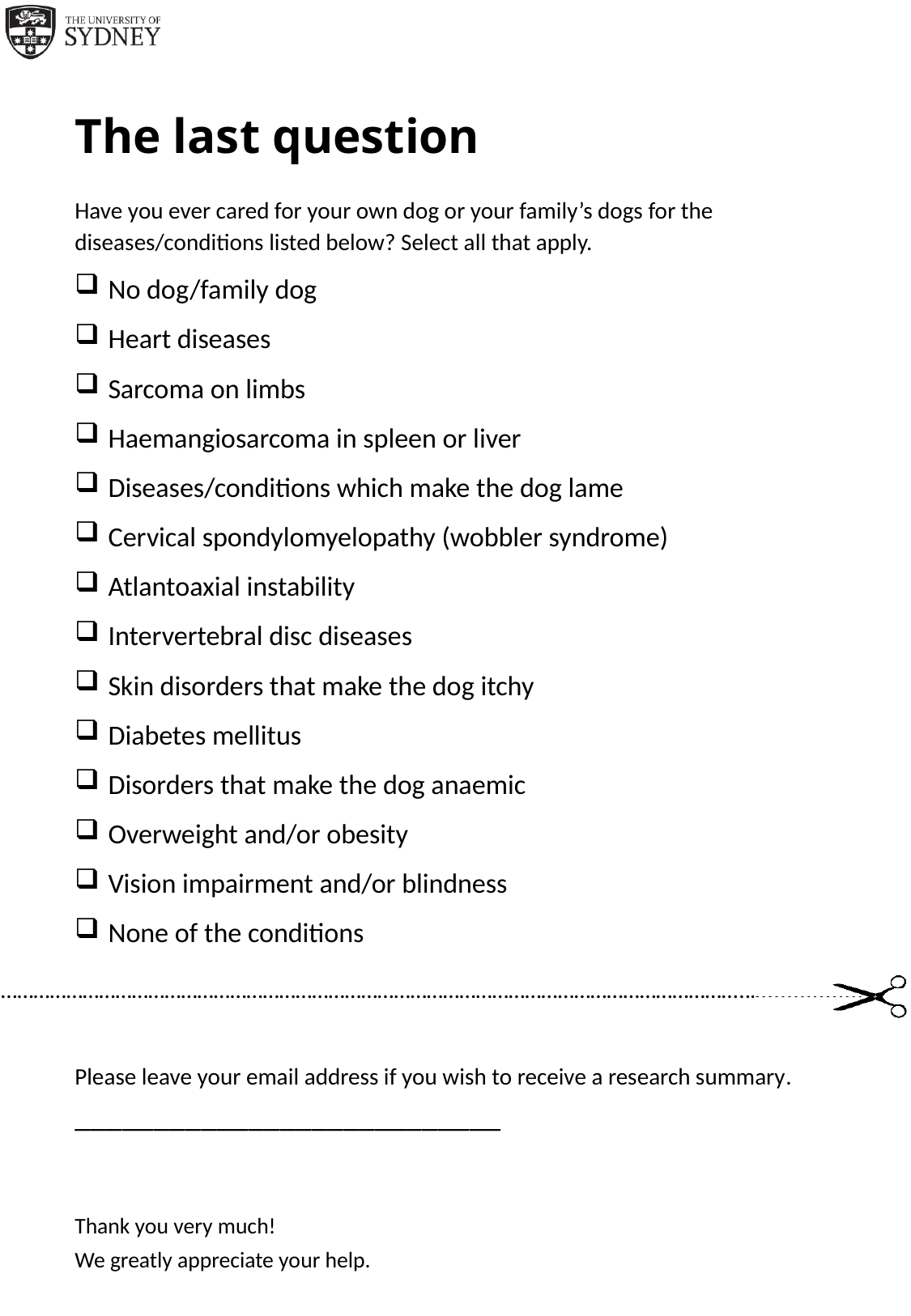

# The last question
Have you ever cared for your own dog or your family’s dogs for the diseases/conditions listed below? Select all that apply.
 No dog/family dog
 Heart diseases
 Sarcoma on limbs
 Haemangiosarcoma in spleen or liver
 Diseases/conditions which make the dog lame
 Cervical spondylomyelopathy (wobbler syndrome)
 Atlantoaxial instability
 Intervertebral disc diseases
 Skin disorders that make the dog itchy
 Diabetes mellitus
 Disorders that make the dog anaemic
 Overweight and/or obesity
 Vision impairment and/or blindness
 None of the conditions
Please leave your email address if you wish to receive a research summary.
___________________________
Thank you very much!
We greatly appreciate your help.
………………………………………………………………………………………………………………………..…
