# Supplementary material for: Welfare-Adjusted Life Years (WALY): A novel metric of animal welfare that combines the impacts of impaired welfare and abbreviated lifespan
Source: PLoS One. 2018 Sep 12;13(9):e0202580. doi: 10.1371/journal.pone.0202580 (PMC6135394; doi:10.1371/journal.pone.0202580)
Supplement: S1 Table — (DOCX) [file pone.0202580.s002.docx]

| **Mild-moderate heart failure (with treatment)** | | |
| --- | --- | --- |
| **Lay description** | After longer periods of intense physical activity, Dog X is more likely than usual to experience tiredness, weakness and laboured breathing. As such, the dog exercises and plays somewhat less. Also, the dog sometimes coughs. | |
|  | **Welfare compromises affecting ≥ 50% of the cases** | **Welfare compromises affecting ≥ 10% but <50% of the cases** |
| **Nutrition** | - Sodium-restricted diet is not provided. |  |
| **Environment** | - Cold weather and night can exacerbate the coughing. | - Hot and humid weather exacerbates the respiratory signs of heart failure. |
| **Health** | - Mild to moderate heart failure  - Some decrease in capacity to endure intense physical activities (e.g. exercise, running, fetching and playing)  - Coughing, particularly during and after longer periods of intense physical activities  - Laboured breathing after longer periods of intense physical activities, such as extended exercise and longer walks. | - Weight loss  - Laboured breathing after moderate exercise or moderate walks  - Ascites  - Syncope |
| **Behaviour** | - Undertakes somewhat fewer intense physical activities than usual  - Somewhat less interaction with people, other dogs or animals than usual | - Eats less than usual; refuses regular food  - Cannot sleep well due to coughing or trouble breathing during the night  - Physical restriction is enforced by the carer*: the dog exercises, plays and interacts with people, other dogs or animals less than usual |
| **Affective state** | - Some discomfort  - Appears tired and weak more easily than usual after longer periods of intense physical activities  - Shortness of breath after longer periods of intense physical activities | - Some loss of appetite  - Fewer positive affective states due to physical restriction than usual  - Sometimes restless at night |
| **Severe heart failure (with treatment)** | | |
| **Lay description** | Dog X is less active and eats less than usual, appears tired and uncomfortable. The dog’s breathing is laboured after moderate levels of physical activity, so the dog exercises, plays and interacts with people or other animals less than usual. The dog coughs and is sometimes restless at night. | |
|  | **Welfare compromises affecting ≥ 50% of the cases** | **Welfare compromises affecting ≥ 10% but <50% of the cases** |
| **Nutrition** | - Sodium-restricted diet is not provided. | - Under-nutrition |
| **Environment** | - Hot and humid weather exacerbates the respiratory signs of heart failure.  - Cold weather and night can exacerbate coughing.  - Soft bedding is needed for long periods of rest. | - Limited access to elevated furniture and stairs (e.g. couch and bed) prevents the dog from accessing places it wants to reach. |
| **Health** | - Severe heart failure  - Decrease in capacity to endure intense physical activities  - Coughing, particularly during and after intense physical activities (e.g. exercise, running, fetching and playing)  - Laboured breathing after moderate exercise or walks | - Syncope  - Vomiting  - Cardiac cachexia |
| **Behaviour** | - Less active than usual  - Undertakes fewer intense physical activities than usual due to physical restriction enforced by the carer* or inability to endure.  - Has less interaction with people, other dogs or animals than usual  - Sometimes eats less than usual; refuses regular food  - Sometimes cannot sleep well due to coughing, particularly during the night | - Does not eat  - Polyuria and polydipsia, which increases urination accidents (i.e. urinates at inappropriate locations)  - Cannot sleep well at night due to trouble breathing |
| **Affective state** | - Tiredness  - Discomfort  - Appears tired and weak more easily than usual during intense physical activities  - Shortness of breath during physical activity  - Sometimes restless at night  - May be frustrated with the affective states listed above  - Fewer positive affective states than usual due to the decrease in exercise, playing and positive interaction with people and other animals | - Anxious about urinating in areas it has been trained to avoid and/or not being able to urinate in the usual places - Thirst  - May be frustrated with not always being able to access to the places where it wants to reach |
| **Cancer: diagnosis and primary therapy** | | |
| **Lay description** | Dog X is less active than usual, appears tired and uncomfortable. The dog exercises, plays and interacts with people or other animals less than usual. The dog eats less than usual and may vomit and/or feels nauseous. | |
|  | **Welfare compromises affecting ≥ 50% of the cases** | **Welfare compromises affecting ≥ 10% but <50% of the cases** |
| **Nutrition** |  |  |
| **Environment** |  | - Cannot access some areas due to vomiting or diarrhoea |
| **Health** | - Neoplastic disease that has not metastasised  - Rapidly growing cells are killed by chemotherapy or radiation  - Weight loss  - Decrease in capacity to endure intense physical activities (e.g. exercise, running, fetching and playing) | - Vomiting and retching  - Diarrhoea  - Lean body condition  - Infection or fever due to a low level of white blood cell  - Anaemia  - Allergic Reactions |
| **Behaviour** | - Eats less than usual; refuses regular food  - Less active than usual  - Undertakes fewer intense physical activities than usual  - Has less interaction with people, other dogs or animals than usual | - Does not eat  - Polyuria and polydipsia, which increases urination accidents (i.e. urinates at inappropriate locations)  - Eats more than usual due to the medication |
| **Affective state** | - Tiredness  - Discomfort  - Appears tired and weak more easily than usual during intense physical activities  - May be frustrated with the affective states listed above  - Fewer positive affective states due to the decrease in exercise, playing and positive interaction with people and other animals than usual  - Some loss of appetite | - Pain  - Nausea  - Loss of appetite  - Hunger  - Thirsty  - Anxious about urinating in areas it has been trained to avoid and/or not being able to urinate in the usual places |
| **Cancer: lung metastasis with/without having metastasis in other parts of the body** | | |
| **Lay description** | Dog X has cachexia, appears tired and uncomfortable, and frequently has laboured breathing. The dog exercises and plays much less, eats and interacts with people or other animals less than usual. The dog is sometimes restless at night due to trouble breathing, coughing and pain. | |
|  | **Welfare compromises affecting ≥ 50% of the cases** | **Welfare compromises affecting ≥ 10% but <50% of the cases** |
| **Nutrition** | - Undernutrition |  |
| **Environment** | - Dust and cigarette smoking in the environment make the dog have more trouble breathing than usual.  - Hot and humid weather makes the dog more likely to have trouble breathing than usual.  - Cold weather makes the dog cough more.  - Soft bedding is needed for long periods of rest.  - Limited access to elevated furniture and stairs (e.g. couch and bed) prevents the dog from accessing places it wants to reach. |  |
| **Health** | - Neoplastic disease that has metastasised to lungs  - Coughing  - Considerable decrease in capacity to endure intense physical activities (e.g. exercise, running, fetching and playing)  - Frequent laboured breathing  - Cachexia  - Chronic anaemia |  |
| **Behaviour** | - Eats less than usual; refuses regular food  - Less active than usual  - Undertakes much fewer intense physical activities than usual  - Has less interaction with people, other dogs or animals than usual  - Cannot sleep and rest well due to trouble breathing, coughing and pain sometimes | - Does not eat |
| **Affective state** | - Some loss of appetite  - Frequent shortness of breath  - Tiredness  - Discomfort  - Sometimes restless at night  - May be frustrated with the affective states listed above and/or not always being able to access to the places where it wants to reach  - Fewer positive affective states than usual due to the decrease in exercise, playing and positive interaction with people and other animals | - Pain  - Entire loss of appetite |
| **Musculoskeletal problems: one limb, mild** | | |
| **Lay description** | Dog X has some pain in one limb, which causes some difficulty in running, walking a long distance, and getting up and lying down. The dog exercises and plays less than usual. | |
|  | **Welfare compromises affecting ≥ 50% of the cases** | |
| **Nutrition** |  | |
| **Environment** |  | |
| **Health** | - Mild functional impairment on one limb: some difficulty in running, walking a long distance and getting up and down  - Some has lameness | |
| **Behaviour** | - Undertakes fewer intense physical activities (e.g. exercise, running, fetching and playing) than usual than usual | |
| **Affective state** | - Some pain  - May be somewhat frustrated with the pain  - Somewhat fewer positive affective states due to the decrease in exercise and playing than usual | |
| **Musculoskeletal problems: one limb, moderate** | | |
| **Lay description** | Dog X has pain in one limb, which causes difficulty in walking, getting up and lying down, accessing some places it wants to reach and even sleeping. The dog exercises and plays much less and eats less than usual. | |
|  | **Welfare compromises affecting ≥ 50% of the cases** | |
| **Nutrition** | - is less willing to approach food and water than usual | |
| **Environment** | - Requires more effort to climb up and down stairs than usual  - Limited access to elevated furniture and stairs (e.g. couch and bed) prevents the dog from accessing places it wants to reach.  - Soft bedding is needed for long periods of rest. | |
| **Health** | - Moderate functional impairment on one limb: has difficulty in running, walking and getting up and down  - Lameness | |
| **Behaviour** | - Less active than usual  - Undertakes much fewer intense physical activities (e.g. exercise, running, fetching and playing) than usual  - Avoids jumping  - Has less interaction with people, other dogs or animals than usual; plays much less than usual  - Cannot sleep and rest well due to the pain sometimes  - Eats less than usual | |
| **Affective state** | - Pain  - May be frustrated with the pain and/or the limited mobility  - Fewer positive affective states than usual due to the decrease in exercise, playing and positive interaction with people and other animals  - Some loss of appetite due to the pain  - Less willing to approach food and water than usual, leaving the dog hungry and thirsty | |
| **Musculoskeletal problems: one limb, severe** | | |
| **Lay description** | Dog X has considerable pain in one limb, making the dog inactive and restless at night. The dog avoids movements that cause further pain (e.g. getting-up and walking), eats and interacts with people or other animals much less than usual and does not exercise or play. | |
|  | **Welfare compromises affecting ≥ 50% of the cases** | |
| **Nutrition** | - Almost unwilling to approach food and water if they are not close | |
| **Environment** | - Soft bedding is needed for long periods of rest.  - An external force (e.g. being touched and being forced to move) may lead to pain. | |
| **Health** | - Severe limb functional impairment  - Severe lameness  - Muscle atrophy | |
| **Behaviour** | - Inactive  - Avoids movements that cause pain, including getting up and down and most locomotor activity.  - Has much less interaction with people, other dogs or animals than usual; does not play  - Cannot sleep and rest well due to the pain  - Eats much less than usual; does not eat | |
| **Affective state** | - High level of pain  - Restless at night  - Tiredness due to insufficient sleep  **-** Hungry and thirsty if the food and water are not accessible  - May be frustrated with the affective states listed above  - Much fewer positive affective states due to the decrease in exercise, playing and positive interaction with people and other animals than usual  - Fear of experiencing pain; irritable or unwilling to be touched  - (Some) loss of appetite due to the pain | |
| **Severe vision impairment and blindness** | | |
| **Lay description** | Dog X is (almost) blind, which makes it harder to undertake various activities, such as running and fetching. Often, the dog cannot fully relax and appears anxious when encountering changes and unknown environments. Sometimes the dog injures itself. | |
|  | **Welfare compromises affecting ≥ 50% of the cases** | |
| **Nutrition** | - Not able to find food and water readily if the location has been changed | |
| **Environment** | - Changes and unknown environments are stressors for the dog  - More likely to get injured by objects in the environment than usual | |
| **Health** | - Severe vision impairment or blindness caused by some health condition(s)  - Injures itself due to not able to see (clearly) | |
| **Behaviour** | - It takes the dog longer to socialise with humans, dogs and other animals.  - It is harder to do some activity, such as running in open space and fetching. | |
| **Affective state** | - Hunger and thirst if the dog cannot find the food and water  - Pain from the injuries  - Cannot be fully relaxed and is anxious when encountering changes and unknown environments.  - Can be startled easily | |
| **Overweight** | | |
| **Lay description** | Dog X is overweight and, therefore, tires rather more easily after high levels of exercise and playing than ideal-weight dogs. | |
|  | **Welfare compromises affecting ≥ 50% of the cases** | **Welfare compromises affecting ≥ 10% but <50% of the cases** |
| **Nutrition** |  | - Decrease in the amount of food given by the carer* |
| **Environment** |  |  |
| **Health** | - Overweight |  |
| **Behaviour** |  |  |
| **Affective state** | - Somewhat appears tired more easily than usual after periods of intense physical activities (e.g. exercise, running, fetching and playing) | - Less satiety than usual |
| **Obesity** | | |
| **Lay description** | Dog X is obese which makes the dog less agile, easier to get tired when exercising and playing and less heat tolerant than slimmer dogs. | |
|  | **Welfare compromises affecting ≥ 50% of the cases** | **Welfare compromises affecting ≥ 10% but <50% of the cases** |
| **Nutrition** |  | - Decrease in the amount of food given by the carer* |
| **Environment** | - Less heat tolerant than ideal-weight dogs |  |
| **Health** | - Obesity |  |
| **Behaviour** | - Less agile in movement than ideal-weight dogs |  |
| **Affective state** | - Appears tired more easily than usual when doing intense physical activities (e.g. exercise, running, fetching and playing) | - Less satiety than usual |
| **Spinal hyperesthesia: mild** | | |
| **Lay description** | Dog X has some neck or back pain, which makes the dog less active than usual. The dog avoids movements that cause pain (e.g. jumping, running and exercise) and plays and eats less than usual. | |
|  | **Welfare compromises affecting ≥ 50% of the cases** | **Welfare compromises affecting ≥ 10% but <50% of the cases** |
| **Nutrition** |  | - Not willing to take food and water on the ground due to cervical pain |
| **Environment** | - External stimuli that make the muscles around the lesion contract cause some pain  - Places requiring jumping or climbing up or down (e.g. couch and bed) become hard for the dog to access |  |
| **Health** | - Mild spinal hyperaesthesia associated with some disorder(s) |  |
| **Behaviour** | - Less active than usual; avoids movement cause pain, such as jumping and running  - Undertakes fewer intense physical activities (e.g. exercise, running, fetching and playing) than usual  - Has less interaction with people, other dogs or animals than usual; plays less than usual  - Eats less than usual | - Cannot sleep and rest well at night |
| **Affective state** | - Pain  - May be frustrated with the pain and the limited mobility  - Fewer positive affective states than usual due to the decrease in exercise, playing and positive interaction with people and other animals  - Some loss of appetite | - Hungry and thirsty when the dog is not willing to approach food and water  - Restless at night, resulting in some tiredness |
| **Spinal hyperesthesia: more severe** | | |
| **Lay description** | Dog X has considerable neck or back pain. The dog avoids movements that cause further pain (e.g. walking and even changing position). The dog does not exercise and play, eats much less than usual, and does not sleep well. The dog appears tired and fearful of being touched. | |
|  | **Welfare compromises affecting ≥ 50% of the cases** | **Welfare compromises affecting ≥ 10% but <50% of the cases** |
| **Nutrition** | - Unwilling to approach food and water  - Not willing to take food and water on the ground due to pain in neck. |  |
| **Environment** | - An external force (e.g. being touched and being forced to move) leads to pain.  - Places requiring jumping or climbing up or down (e.g. couch and bed) are almost impossible for the dog to access | - Odour in the environment due to not being able to move elsewhere to urinate/soil |
| **Health** | - Severe spinal hyperaesthesia associated with some disorder(s)  - Muscle rigidity |  |
| **Behaviour** | - Avoids various movements that cause pain, such as getting up and down, locomotory activity and even changing position.  - Has much less interaction with people, other dogs or animals than usual; does not play  - Cannot sleep and rest well due to the pain  - Eats much less than usual; does not eat |  |
| **Affective state** | - High level of pain  - Restless at night, resulting in some tiredness  - Hungry and thirsty when the dog is not willing to approach food and water  - May be frustrated with the affective states listed above and the limited mobility  - Much fewer positive affective states due to the decrease in exercise, playing and positive interaction with people and other animals than usual  - Fear of experiencing pain; irritable or unwilling to be touched  - (Some) loss of appetite | - Anxious about urinating/soiling in areas it has been trained to avoid and/or not being able to urinate/soiling in the usual places |
| **Ataxia, paraparesis or tetraparesis** | | |
| **Lay description** | Dog X cannot walk and run as well as it did before and is likely to feel weak on its limbs due to ataxia or paresis. This makes exercising, socialising, playing and accessing places it wants to reach more difficult than usual. | |
|  | **Welfare compromises affecting ≥ 50% of the cases** | **Welfare compromises affecting ≥ 10% but <50% of the cases** |
| **Nutrition** | - More effort is needed to approach food and water than usual |  |
| **Environment** | - Limited access to elevated furniture and stairs (e.g. couch and bed) prevents the dog from accessing places it wants to reach.  - More likely to get injured by objects in the environment than usual |  |
| **Health** | - Ataxia, ambulatory paraparesis or ambulatory tetraparesis caused by some health condition(s)  - Muscle weakness | -Injures itself due to poor control of the body |
| **Behaviour** | - Not able to move its limbs as it did before: harder to do intense physical activities (e.g. exercise, running, fetching and playing), socialise with other dogs and access to the places it wants to reach; some loss of autonomy |  |
| **Affective state** | - May be confused and/or frustrated with the muscle weakness and some loss of autonomy | - Some pain from the injuries |
| **Non-ambulatory paraparesis or paraplegia** | | |
| **Lay description** | Dog X‘s hindlimbs are not ambulatory due to paraparesis or paraplegia. The dog is not able to exercise and play, and it’s hard to access the places it wants to reach and to interact with people or other animals for the dog. | |
|  | **Welfare compromises affecting ≥ 50% of the cases** | **Welfare compromises affecting ≥ 10% but <50% of the cases** |
| **Nutrition** | - More effort is needed to approach food and water than usual |  |
| **Environment** | - Limited access to elevated furniture and stairs (e.g. couch and bed) prevents the dog from accessing places it wants to reach.  - Soft bedding is needed for long periods of rest. | -Urinary odour in the environment due to not being able to move elsewhere to urinate |
| **Health** | - None-ambulatory paraparesis or paraplegia caused by some health condition(s)  - Drags hindlimbs which causes some wounds  - Muscle atrophy  - Joint stiffness in hindlimbs | - Decubitus  - Urine scald |
| **Behaviour** | - Not able to move the hindlimbs: is much harder to move around, and access to the places it wants to reach and not able to do intense physical activities; much loss of autonomy  - Has much less interaction with people, other dogs or animals than usual |  |
| **Affective state** | - Some pain from dragging the hindlimbs, wounds and joint stiffness are present  - Hungry and thirsty when the food and water is too hard to be approached  - May be frustrated with the affective states listed above and the loss of autonomy  - May be confused and/or frustrated with not being able to move the hindlimbs  - Much fewer positive affective states due to the decrease in exercise, playing and positive interaction with people and other animals than usual | - Pain from decubitus  - Skin irritation due to contact with urine  - Anxious about urinating/soiling in areas it has been trained to avoid and/or not being able to urinate/soiling in the usual places |
| **Non-ambulatory paraparesis or paraplegia: on wheelchair some hours a day** | | |
| **Lay description** | Dog X‘s hindlimbs are not ambulatory, so it is in a wheelchair for some hours a day. This helps the dog walking, exercising and interacting with people or other animals to a certain extent. During the hours without a wheelchair, the dog has very limited mobility. | |
|  | **Welfare compromises affecting ≥ 50% of the cases** | **Welfare compromises affecting ≥ 10% but <50% of the cases** |
| **Nutrition** |  |  |
| **Environment** | - Limited access to elevated furniture and stairs (e.g. couch and bed) prevents the dog from accessing places it wants to reach.  - Soft bedding is needed for long periods of rest. |  |
| **Health** | - Non-ambulatory paraparesis or paraplegia caused by some health condition(s).  - Redness or wounds on skin due to rubbing against the wheelchair  - Muscle strain and soreness caused by using a wheelchair  - Muscle atrophy  - Joint stiffness in hindlimbs | - Decubitus  - Drags hindlimbs which causes some wounds |
| **Behaviour** | - The mobility and autonomy of the dog are improved by the wheelchair but still limited; the dog can walk and even exercise and play to some extent. |  |
| **Affective state** | - Some pain resulting from joint stiffness  - May be confused and/or frustrated with not being able to move the hindlimbs  - Somewhat fewer positive affective states due to the decrease in exercise, playing and positive interaction with people and other animals than usual | - Pain from decubitus and wounds |
| **Non-ambulatory tetraparesis or tetraplegia** | | |
| **Lay description** | Dog X’s four limbs are not ambulatory due to neurological condition(s). The dog cannot move, approach food and water, exercise, play, access the places it wants to reach and interact with people or other animals. The dog may have a skin irritation caused by urine scald. | |
|  | **Welfare compromises affecting ≥ 50% of the cases** | **Welfare compromises affecting ≥ 10% but <50% of the cases** |
| **Nutrition** | - Cannot approach food and water |  |
| **Environment** | - Soft bedding is needed for long periods of rest.  - Odour in the environment due to not being able to move elsewhere to urinate/soil |  |
| **Health** | - Non-ambulatory tetraparesis or tetraplegia caused by some health condition(s)  - Muscle atrophy  - Joint stiffness in four limbs  - Urine scald | - Decubitus |
| **Behaviour** | - Cannot move and do most physical activity; loss of autonomy  - Has much less interaction with people, other dogs or animals than usual |  |
| **Affective state** | - Hunger and thirst if the carer* does not make food and water accessible to the dog  - Some pain from joint stiffness  - Skin irritation due to contact with urine  - May be frustrated with the affective states listed above and the loss of autonomy  - May be confused and/or frustrated with not being able to move the limbs  - Much fewer positive affective states due to the decrease in exercise, playing and positive interaction with people and other animals than usual  - Anxious about urinating/soiling in areas it has been trained to avoid and/or not being able to urinate/soiling in the usual places | - Pain from decubitus |
| **Urinary incontinence (upper motor neuron)** | | |
| **Lay description** | Dog X cannot urinate voluntarily, so bladder expression by the carer* is needed twice a day, causing some discomfort and pain. The dog is restless when the bladder is particularly distended and sometimes has a skin irritation caused by urine scald. | |
|  | **Welfare compromises affecting ≥ 50% of the cases** | **Welfare compromises affecting ≥ 10% but <50% of the cases** |
| **Nutrition** |  |  |
| **Environment** |  | -Urinary odour in the environment |
| **Health** | - Upper motor neuron bladder dysfunction caused by impaired spinal cord  - Urine retention  - Urine leakage when too much urine in the bladder  - Urine scald | - Urinary tract infection |
| **Behaviour** | - Cannot urinate voluntarily |  |
| **Affective state** | - Discomfort and some pain due to extended bladder and bladder expression by the carer*  - Restless due to extended bladder  - A skin irritation caused by urine scald | - Discomfort and pain caused by urinary tract infection  - Vesical tenesmus caused by urinary tract infection |
| **Urinary incontinence (lower motor neuron)** | | |
| **Lay description** | Dog X is unable to store and void urine normally, so the urine often drips, resulting in a skin irritation. Bladder expression by the carer* is needed many times a day, causing discomfort and pain. The dog has repeated urinary tract infections causing general discomfort and pain when urinating. | |
|  | **Welfare compromises affecting ≥ 50% of the cases** | |
| **Nutrition** |  | |
| **Environment** | - Urinary odour in the environment | |
| **Health** | - The bladder is unable to store and void urine normally  - Urine leakage  - Urine retention  - Urinary tract infection  - Urine scald | |
| **Behaviour** | - Has less interaction with people than usual due to the smell of urine | |
| **Affective state** | - Discomfort and/or some pain due to bladder expression by the carer*  - Discomfort and pain caused by urinary tract infection  - Vesical tenesmus caused by urinary tract infection  - A skin irritation caused by urine scald  - May be frustrated with the affective states listed above. | |
| **Anaemia: mild** | | |
| **Lay description** | Dog X sometimes appears tired and weak after exercise and playing, but this does not interfere with daily life. | |
|  | **Welfare compromises affecting ≥ 50% of the cases** | **Welfare compromises affecting ≥ 10% but <50% of the cases** |
| **Nutrition** |  |  |
| **Environment** |  |  |
| **Health** | - Mild anaemia caused by some health condition(s) | - Some muscle weakness |
| **Behaviour** |  |  |
| **Affective state** |  | - Some tiredness  - Some weakness |
| **Anaemia: moderate** | | |
| **Lay description** | Dog X is less active than usual and appears tired and weak. The dog can be short of breath after intense physical activities, so the dog exercises, plays and interacts with people or other animals less than usual. The dog eats less than usual. | |
|  | **Welfare compromises affecting ≥ 50% of the cases** | |
| **Nutrition** |  | |
| **Environment** |  | |
| **Health** | - Moderate anaemia caused by some health condition(s)  - Some muscle weakness  - Laboured breathing and/or rapid breathing after extended intensive physical activity  - Decrease in capacity to endure intense physical activities (e.g. exercise, running, fetching and playing) | |
| **Behaviour** | - Less active than usual  - Undertakes fewer intense physical activities than usual  - Has less interaction with people, other dogs or animals than usual; plays less  - Eats less than usual | |
| **Affective state** | - Tiredness  - Weakness  - Shortness of breath after some intense physical activities  - May be frustrated with the affective states listed above  - Fewer positive affective states than usual due to the decrease in exercise, playing and positive interaction with people and other animals  - Some loss of appetite | |
| **Anaemia: severe** | | |
| **Lay description** | Dog X often has laboured breathing and appears tired and weak. The dog does not exercise and play, has little interaction with people or other animals and eats much less than usual. | |
|  | **Welfare compromises affecting ≥ 50% of the cases** | |
| **Nutrition** |  | |
| **Environment** | - Hot and humid weather makes the dog more likely to have trouble breathing than usual.  - Limited access to elevated furniture and stairs (e.g. couch and bed) prevents the dog from accessing places it wants to reach.  - Soft bedding is needed for long periods of rest. | |
| **Health** | - Severe anaemia caused by some health condition(s)  - Muscle weakness  - Laboured and/or rapid breathing  - Weight loss | |
| **Behaviour** | - Much less active than usual  - Does not undertake intense physical activities (e.g. exercise, running, fetching and playing)  - Has much less interaction with people, other dogs or animals than usual; plays much less than usual  - Eats much less than usual, refuses regular food or does not eat | |
| **Affective state** | - Tiredness  - Weakness  - Short of breath  - May be frustrated with the affective states listed above  - Much fewer positive affective states due to the decrease in exercise, playing and positive interaction with people and other animals than usual  - Loss of appetite | |
| **Pruritus and discomfort: mild** | | |
| **Lay description** | Dog X has some itchiness, but not when sleeping, eating, playing, exercising or being otherwise distracted. The dog spends more time scratching and/or chewing than usual. | |
|  | **Welfare compromises affecting ≥ 50% of the cases** | |
| **Nutrition** |  | |
| **Environment** |  | |
| **Health** | - Skin lesions | |
| **Behaviour** | - Over scratching  - Over chewing | |
| **Affective state** | - Some itchiness during the day, but not when sleeping, eating, playing, exercising or being distracted. | |
| **Pruritus and discomfort: moderate** | | |
| **Lay description** | Dog X often remains itchy, even during sleep, but not when eating, playing or being otherwise distracted. The dog spends moderate amounts of time scratching and/or chewing itself, causing wounds with/without secondary infection. The dog cannot sleep too well. | |
|  | **Welfare compromises affecting ≥ 50% of the cases** | |
| **Nutrition** |  | |
| **Environment** |  | |
| **Health** | - Skin lesions  - Some wounds with or without secondary infection | |
| **Behaviour** | - Over scratching, even during sleeping  - Over chewing, even during sleeping  - Cannot sleep too well due to the itchiness | |
| **Affective state** | - Itchiness during the day and while sleeping, but not when eating, playing, exercising or being distracted  - Restless at night, resulting in some tiredness  - Some skin irritation due to the wounds  - May be frustrated with the affective states listed above | |
| **Pruritus and discomfort: severe** | | |
| **Lay description** | Dog X is itching during the day and night, as well as when eating, playing or being distracted. The dog is restless and spends excessive amounts of time scratching and/or chewing itself which affects its other daily activities and causes some wounds with/without secondary infection. | |
|  | **Welfare compromises affecting ≥ 50% of the cases** | |
| **Nutrition** |  | |
| **Environment** |  | |
| **Health** | - Skin lesions  - Some wounds with or without secondary infection | |
| **Behaviour** | - Over scratching that has affected daily activity, such as eating, exercising and socialising.  - Over chewing that has affected daily activity, such as eating, exercising and socialising.  - Cannot sleep well due to the itchiness | |
| **Affective state** | - Itchiness during the day and while sleeping, as well as when eating, playing, exercising or being distracted  - Restless at night, resulting in tiredness  - Some skin irritation due to the wounds  - May be frustrated with the affective states listed above  - Fewer positive affective states because the dog cannot concentrate on exercise, playing and positive interaction with people and other animals as much as usual | |
| **Respiratory distress** | | |
| **Lay description** | Dog X has respiratory distress. The dog avoids moving its body and has almost no interaction with people or other animals. The dog does not eat or drink (or drinks much less than usual) and appears tired. | |
|  | **Welfare compromises affecting ≥ 50% of the cases** | **Welfare compromises affecting ≥ 10% but <50% of the cases** |
| **Nutrition** |  |  |
| **Environment** | - Hot and humid weather makes the dog more likely to have trouble breathing than usual.  - External stimuli may disturb the dog’s breathing |  |
| **Health** | - Laboured breathing caused by some health condition(s)  - Muscle weakness |  |
| **Behaviour** | - Does not eat  - Drinks less water than usual or does not drinks water  - Avoids movements that interfere breathing, including most locomotor activity and even changing position.  - Has almost no interaction with people, other dogs and animals  - Cannot sleep and rest well |  |
| **Affective state** | - Tiredness  - Weakness  - Shortness of breath and the dog may be frustrated with that  - Much fewer positive affective states than usual due to the decrease in exercise, playing and positive interaction with people and other animals  - Thirst |  |
| **Polyuria and polydipsia** | | |
| **Lay description** | Dog X urinates and drinks more than usual. The dog is thirsty and appears anxious when not being able to urinate in usual places. | |
|  | **Welfare compromises affecting ≥ 50% of the cases** | **Welfare compromises affecting ≥ 10% but <50% of the cases** |
| **Nutrition** |  |  |
| **Environment** | - Urinary odour in the environment |  |
| **Health** | - Polyuria and polydipsia due to some health condition(s) | - Urinary incontinence due to too much urine |
| **Behaviour** | - Drinks more water than usual  - Urinates more | - Urination accidents are increased (i.e. urinates at inappropriate locations)  - Urine scald |
| **Affective state** | - Thirst  - Anxious about urinating in areas it has been trained to avoid and/or not being able to urinate in the usual places | - Skin irritation due to contact with urine |
| **Vomiting: two times or more a day** | | |
| **Lay description** | Dog X vomits two or more times a day and has some nausea and/or abdominal discomfort. The dog eats less than usual or not at all and drinks less water than usual. | |
|  | **Welfare compromises affecting ≥ 50% of the cases** | **Welfare compromises affecting ≥ 10% but <50% of the cases** |
| **Nutrition** |  | - Under-nutrition |
| **Environment** |  |  |
| **Health** | - Vomiting and retching | - Dehydration  - Weight loss  - Lean body condition |
| **Behaviour** | - Eats less than usual; does not eat  - Drinks less water than usual |  |
| **Affective state** | - (Some) loss of appetite  - Nausea  - Discomfort due to vomiting and retching  - Abdominal discomfort or pain  - Thirst | - Weakness |
| **Diarrhoea: two times or more a day** | | |
| **Lay description** | Dog X has diarrhoea two or more times a day and drinks more water than usual because of dehydration and thirst. | |
|  | **Welfare compromises affecting ≥ 50% of the cases** | **Welfare compromises affecting ≥ 10% but <50% of the cases** |
| **Nutrition** |  | - Under-nutrition |
| **Environment** |  |  |
| **Health** | - Diarrhoea  - Dehydration | - Weight loss  - Lean body condition |
| **Behaviour** | - Drinks more water than usual | - Eats less than usual |
| **Affective state** | - Thirst | - Some loss of appetite  - Rectal tenesmus  - Abdominal discomfort  - Weakness |
| **Abdominal pain or discomfort** | | |
| **Lay description** | Dog X has abdominal pain and/or discomfort. The dog is less active, and exercises, plays and interacts with people or other animals less than usual. | |
|  | **Welfare compromises affecting ≥ 50% of the cases** | **Welfare compromises affecting ≥ 10% but <50% of the cases** |
| **Nutrition** |  |  |
| **Environment** |  |  |
| **Health** | - Some health condition(s) causing abdominal pain or discomfort |  |
| **Behaviour** | - Less active than usual  - Undertakes fewer intense physical activities (e.g. exercise, running, fetching and playing) than usual  - Has less interaction with people, other dogs or animals than usual; plays less than usual  - Almost does not eat |  |
| **Affective state** | - Abdominal pain and/or discomfort  - May be frustrated with the affective states listed above  - Fewer positive affective states than usual due to the decrease in exercise, playing and positive interaction with people and other animals  - (Some) loss of appetite | - Nausea |
| **Lethargy and loss of appetite** | | |
| **Lay description** | Dog X is lethargic and not eating. The dog is less active than usual, and exercises, plays and interacts with people or other animals less than usual. The dog appears to be tired and have some discomfort. | |
|  | **Welfare compromises affecting ≥ 50% of the cases** | |
| **Nutrition** |  | |
| **Environment** |  | |
| **Health** | - Decrease in capacity to endure intense physical activities (e.g. exercise, running, fetching and playing)  - Weight loss | |
| **Behaviour** | - Does not eat  - Less active than usual  - Undertakes fewer intense physical activities than usual  - Less interaction with people, other dogs or animals than usual; plays less than usual | |
| **Affective state** | - Tiredness  - Discomfort  - Loss of appetite | |
| **Fever** | | |
| **Lay description** | Dog X has a fever, does not eat, and appears tired and uncomfortable. The dog is less active, and exercises, plays and interacts with people or other animals less than usual. | |
|  | **Welfare compromises affecting ≥ 50% of the cases** | |
| **Nutrition** |  | |
| **Environment** |  | |
| **Health** | - Fever  - Decrease in capacity to endure intense physical activities (e.g. exercise, running, fetching and playing) | |
| **Behaviour** | - Eats less than usual; does not eat  - Less active than usual  - Undertakes fewer intense physical activities than usual  - Has less interaction with people, other dogs or animals than usual; plays less | |
| **Affective state** | - Tiredness  - Discomfort  - Fewer positive affective states than usual due to the decrease in exercise, playing and positive interaction with people and other animals  - (Some) loss of appetite | |
| **Amputation: one limb** | | |
| **Lay description** | Dog X has lost a part of one limb, leaving three functional limbs. The dog appears to tire more easily than dogs with four limbs when exercise/playing and appears hungry if the carer* practices restrictive diet. | |
|  | **Welfare compromises affecting ≥ 50% of the cases** | **Welfare compromises affecting ≥ 10% but <50% of the cases** |
| **Nutrition** |  | - Same amount of food by the carer* as what was fed before amputation |
| **Environment** | - Stairs and elevated furniture (e.g. couch and bed) require the dog more efforts to climb up and down than dogs having four limbs |  |
| **Health** | - Limb functional impairment: has only three limbs  - More burden on the remaining limbs than dogs having four limbs, which increases the risk for other musculoskeletal conditions  - Some decrease in capacity to endure intense physical activities (e.g. exercise, running, fetching and playing) | - Overweight or obesity |
| **Behaviour** | - Undertakes somewhat fewer intense physical activities than usual |  |
| **Affective state** | - Appears tired and more easily than usual when doing intense physical activities  - Hunger if the carer* feed less than what was fed before amputation |  |
| **Frequent veterinary visits (for fearful dogs)** | | |
| **Lay description** | Dog X visits a veterinarian at least once every two months. The dog shows signs of fear of the event and cannot relax during the visit. | |
|  | **Welfare compromises affecting ≥ 50% of the cases** | |
| **Nutrition** |  | |
| **Environment** | - Veterinary clinic or veterinarians | |
| **Health** |  | |
| **Behaviour** | - Short-term stress response in the clinic, such as panting and trembling  - Restriction of movement during examinations and treatment  - Restriction of fight or flight response | |
| **Affective state** | - Fear  - Stress  - Some pain and discomfort from examinations and treatment  - Cannot fully relax during the visit | |
| **Frequent subcutaneous injections by carers* at home** | | |
| **Lay description** | Dog X receives subcutaneous injection multiple times daily or weekly. The dog avoids or resists the accompanying handling; indicating dislike of injections or fear of the pain caused by injections. | |
|  | **Welfare compromises affecting ≥ 50% of the cases** | **Welfare compromises affecting ≥ 10% but <50% of the cases** |
| **Nutrition** |  |  |
| **Environment** |  |  |
| **Health** |  | - Complications at the injection sites |
| **Behaviour** | - Restriction of movement during the injection | - Hides from the carer* at time injections due |
| **Affective state** | - Some pain from injections  - Dislike of injections  - Fear of the pain | - Soreness or pain from complications at the injection sites |
| **Death** | | |
| Dog X is dead | | |

*Carers could mean owners, animal attendants, trainers or guardians.
